# Facile Single‐Nanocomposite 4D Bioprinting of Dynamic Hydrogel Constructs with Thickness‐Controlled Gradient

**DOI:** 10.1002/advs.202509449

**Published:** 2025-07-14

**Authors:** Jiahui Lai, Tiandi Xiong, Shangsi Chen, Zhilong Zhou, Jun Liu, Boguang Yang, Rocky S. Tuan, Zhong Alan Li

**Affiliations:** ^1^ Department of Biomedical Engineering The Chinese University of Hong Kong Shatin, NT Hong Kong SAR 999077 P. R. China; ^2^ InnoHK Center for Neuromusculoskeletal Restorative Medicine Hong Kong Science Park, NT Hong Kong SAR 999077 P. R. China; ^3^ Institute for Tissue Engineering and Regenerative Medicine School of Biomedical Sciences The Chinese University of Hong Kong Hong Kong SAR 999077 P. R. China; ^4^ Shun Hing institute of Advanced Engineering The Chinese University of Hong Kong Hong Kong SAR 999077 P. R. China

**Keywords:** 4D bioprinting, MXene, smart hydrogels, thickness‐controlled crosslinking gradient, tissue engineering

## Abstract

The advent of 4D bioprinting has fueled significant progress in tissue engineering, but it faces major challenges such as limited options of smart bioinks and complexity of designing printing paths, limiting its broader application in tissue engineering. In this study, a smart composite hydrogel is first developed by combining gelatin, gelatin methacryloyl, and MXene (MX/GG), exhibiting excellent printability and shape‐morphing capabilities. A facile and robust 4D printing strategy is proposed to fabricate MX/GG hydrogels with distinct spatial crosslinking gradients by simply tuning the domain‐specific pattern thickness followed by a single UV exposure. Finite element analysis is applied to effectively guide the thickness‐controlled shape‐morphing process, resulting in precise alignment with the actual curved constructs and reliably predicting the shape transformation of CAD‐designed patterns. Inspired by natural shape‐morphing systems, a wide range of biomimetic constructs are successfully 4D printed, including unidirectional curved constructs (e.g., five‐petal flower) and bidirectional curved constructs (e.g., scorpion). As a proof‐of‐concept, cell‐laden MX/GG bioinks are 4D bioprinted into humidity‐driven self‐folding strips. Living cells experienced bending‐associated strain within 3D constructs and proliferated and functioned effectively. Developed with the facile 4D printing strategy, the MXene‐reinforced smart hydrogels hold significant promise for the biofabrication of diverse programmable dynamic tissues and organs.

## Graphical abstract

This study presents a novel and facile thickness‐modulated 4D bioprinting approach using a smart bioink made of gelatin, GelMA, and MXene. Leveraging computational models, it creates living constructs with programmable unidirectional and bidirectional curvatures under a single UV exposure. Biocompatibility is demonstrated by high cell viability, neurite outgrowth, and vascular formation, highlighting its potential for tissue regeneration.

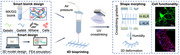

## 1 Introduction

3D bioprinting has significantly enhanced our capability to engineer tissues. However, the dynamic nature of native tissues, which experience spatiotemporal remodeling, necessitates the incorporation of a fourth dimension, time, thus leading to the development of 4D bioprinting. This advanced biofabrication technique enables the creation of cell‐laden constructs that can achieve programmable post‐printing transformations in shape or functionality in response to various stimuli such as water, temperature, and magnetic field.^[^ ^1^, ^2^, ^3^, ^4^ ^]^ Two crucial components of 4D bioprinting are: 1) smart bioinks, which comprise smart hydrogels, biological cues, and living cells; and 2) smart design, which directs the programmable transformation of shape and/or functionality. 4D bioprinting has been applied to create diverse programmable dynamic tissues and organs. For instance, Alsberg's group successfully 4D bioprinted various water‐responsive, cell‐laden constructs utilizing oxidized and methacrylated alginate (OMA) and gelatin methacryloyl (GelMA) via an extrusion‐based bioprinter.^[^ ^5^, ^6^, ^7^, ^8^, ^9^ ^]^ In their strategy, a UV absorber was applied to mediate the crosslinking gradient formation during UV‐induced polymerization. When swollen, these constructs automatically transformed to a bending configuration mimicking the natural curvature of bone and cartilage. This UV absorber‐mediated strategy is effective for building constructs with unidirectional bending but requires an additional photomask for fabricating those with bidirectional deformation, which may complicate the fabrication of bi‐directional curved scaffolds and limit its wider applications. Wang's group performed 4D bioprinting of cell‐laden, shape memory scaffolds with multiple layers, including a shape memory layer made from poly(L‐lactide‐*co*‐trimethylene carbonate) (PLLA‐*co*‐TMC) and a cell‐laden functional layer made from GelMA‐based hydrogels.^[^ ^10^, ^11^, ^12^ ^]^ When heated to human body temperature, these multilayered scaffolds could fold into tubular structures, showing high potential as curved scaffolds for uterine regeneration. However, this strategy based on shape memory polymer (SMP) often requires a multi‐material bioprinting system and a reshaping step for SMP. Such a multi‐step and multi‐material bioprinting process often is complex and have limited scalability. Moreover, these recent applications of 4D bioprinting have been constrained to simple folding behaviors, which are inadequate for recapitulating the intricate curvatures of native organs, such as the multidirectional folding of epithelial tissues.

Smart hydrogels and smart designs are two crucial components toward achieving desirable shape transformation in 4D printing. Humidity‐responsive smart hydrogels typically undergo controlled volumetric changes via solvent exchange, and smart designs spatially orchestrate internal stresses to govern shape change. Several smart hydrogels, such as methylcellulose/alginate,^[^ ^13^, ^14^ ^]^ GelMA,^[^ ^15^, ^16^, ^17^ ^]^ and silk,^[^ ^18^ ^]^ have been applied for 4D bioprinting. However, the availability of smart hydrogels for 4D bioprinting remains limited due to the stringent requirements spanning printability, biocompatibility, robust shape‐morphing capabilities, mechanical and structural integrity, and suitable cell sources.^[^ ^19^, ^20^, ^21^ ^]^ Current smart designs for 4D bioprinting, which can modulate the printed components across the thickness, plane, or both,^[^ ^22^, ^23^ ^]^ frequently struggle to balance simplicity and achieving desired 4D effects. Previously reported strategies typically involved multi‐material gradients or complex biofabrication procedures to achieve complex curvatures,^[^ ^24^, ^25^, ^26^ ^]^ which were time consuming and labor intensive, thereby limiting the scalability of the procedures. Additionally, previously reported 4D bioprinted constructs mostly exhibited unidirectional bending, but few succeeded in replicating the multidirectional curvatures and folding patterns observed in functional native tissues. Overall, as 4D bioprinting is still in its proof‐of‐concept stage, current challenges in this field include limited smart bioinks and specific smart design techniques.^[^ ^27^ ^]^ Thus, the 4D printing field urgently needs smart hydrogels with high printability and robust, versatile shape‐morphing behaviors, along with 4D printing strategies that enable simplified programming of intricate curvatures.

**Figure 1:**
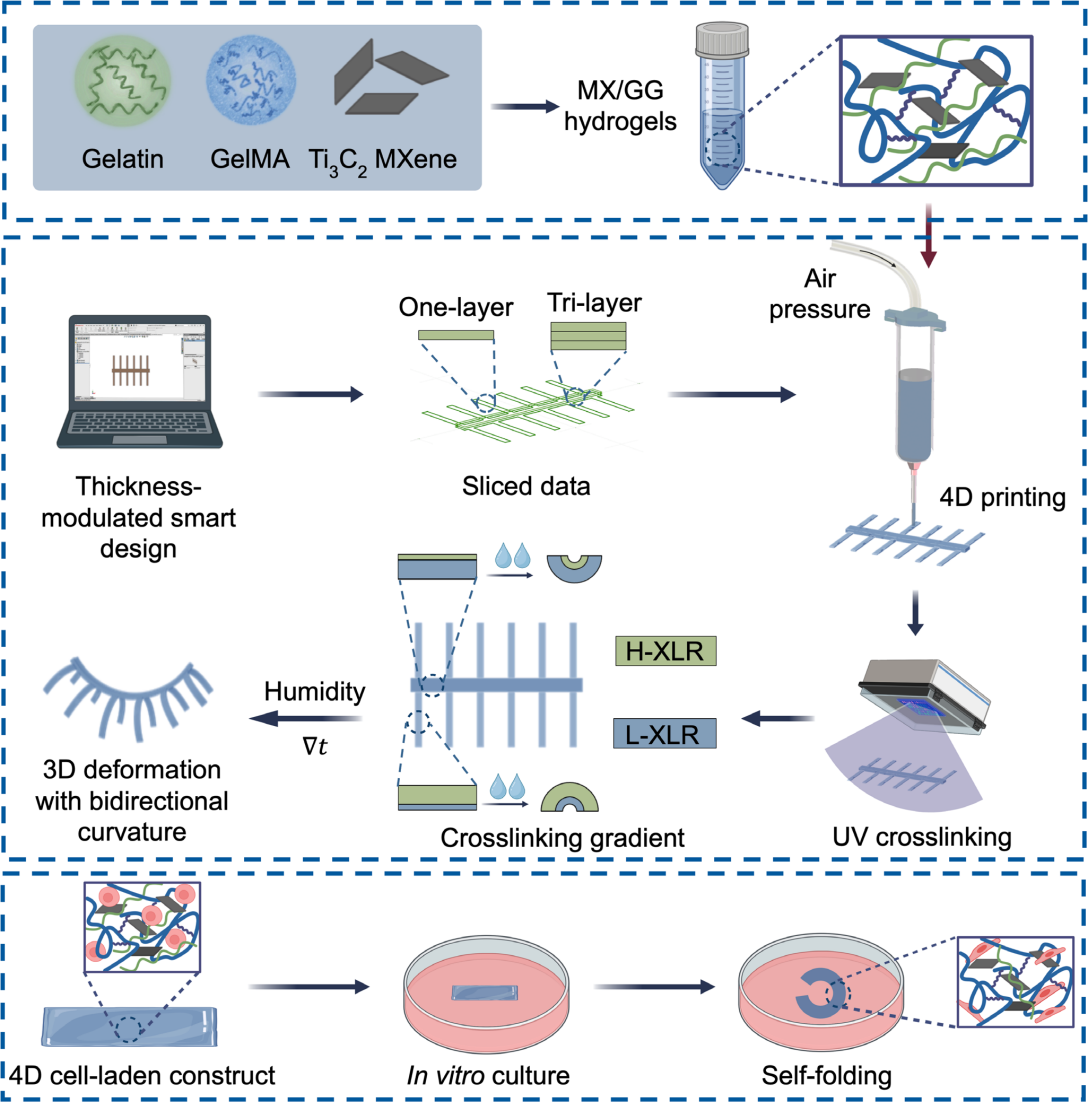
Schematic illustration of 4D bioprinting of cell‐laden MX/GG hydrogels with programmable, humidity‐responsive 3D deformation. H‐XLR: high crosslinking region; L‐XLR: low crosslinking region.

In this study, to mitigate the current challenges of 4D bioprinting, a novel and facile thickness‐based approach was proposed for achieving 4D bioprinting of unidirectional and bidirectional curved constructs with a smart hydrogel integrating GelMA, gelatin, and MXene. While GelMA and gelatin are both biocompatible hydrogels widely applied in 3D bioprinting,^[^ ^28^, ^29^, ^30^, ^31^, ^32^, ^33^, ^34^, ^35^, ^36^ ^]^ their potential for shape morphing has been underexplored, which limits their broader applications in 4D bioprinting. MXene, a 2D transition metal carbide, offers numerous advantages such as excellent electrical conductivity, biocompatibility, and antibacterial properties, making it highly appealing for tissue engineering.^[^ ^37^, ^38^, ^39^, ^40^, ^41^, ^42^ ^]^ Incorporating MXene into GelMA and gelatin bioinks provides multiple benefits, including enhanced electrical conductivity, improved shape morphing capabilities, and a UV shielding effect that protects cells from UV damage. These features, especially improved shape‐morphing ability, make the bioink highly suitable for 4D bioprinting applications. Notably, using the simple approach of a single UV exposure, we generated distinct thickness‐dependent gradients in crosslinking degree at different shape domains. This resulted in spatially anisotropic swelling, which contributes to the shape morphing ability of the printed constructs (**Figure** **1**). Significantly, having demonstrated that the results from finite element analysis (FEA) align well with experimental data, we successfully utilized FEA to guide our smart pattern design and reliably predict the shape morphing behaviors of complex printed constructs, including unidirectional curvature and bidirectional curved morphologies. Furthermore, as proof of concept, we 4D bioprinted constructs incorporating neuronal PC12 cells and human umbilical vein endothelial cells (HUVECs). The dynamic cell‐laden constructs supported neurite outgrowth in encapsulated PC12 cells and promoted the formation of vasculature‐like structures in HUVECs. Our innovative, facile 4D bioprinting strategy obviates the need for multi‐material systems or post‐printing modifications, thereby simplifying the fabrication process of living constructs with programmable bidirectional curvature.

## 2 Results

### 2.1 Characteristics of MXene‐Reinforced Shape Morphing Hydrogels

The synthesized GelMA was characterized via ^1^H NMR. Both ^1^H NMR spectrum and solidification of GelMA solution under UV confirmed the successful synthesis of GelMA (Figure S1, Supporting Information).^[^ ^34^, ^43^ ^]^ The procured MXene was characterized using various methods (Figure S2, Supporting Information), including photographic observation of MXene solutions at different concentration, UV–vis–NIR absorption spectrum (330 to 1,000 nm), X‐ray diffraction, Raman spectroscopy, ultrastructural imaging using transmission (TEM) and scanning electron microscopy (SEM). These results demonstrate the specific absorbance peak and layered structure of MXene nanosheets similar to those in reported literature,^[^ ^37^, ^38^, ^40^, ^44^, ^45^, ^46^ ^]^ verifying the good quality of the obtained MXene.

**Figure 2:**
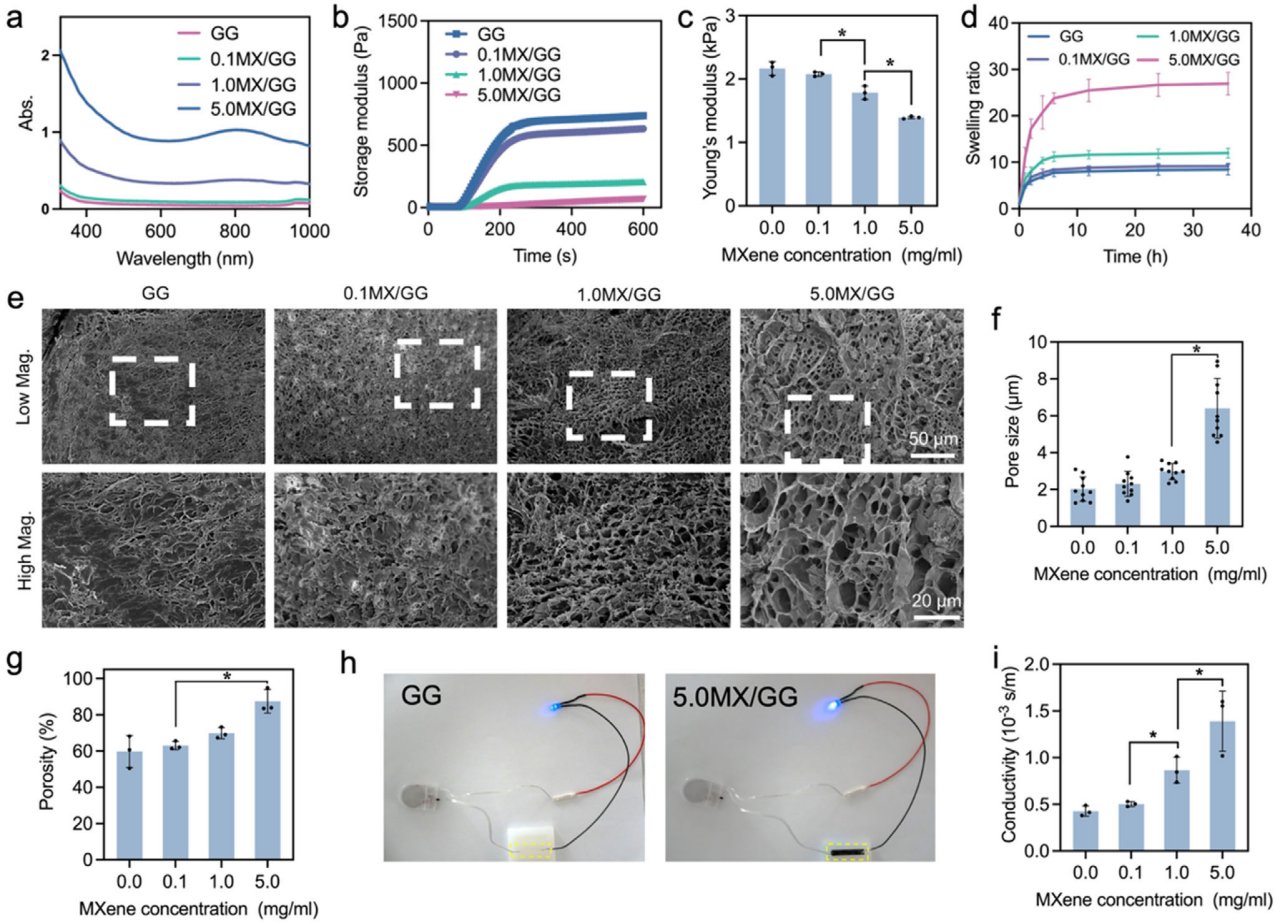
Characterization of MX/GG hydrogels. a) Light absorbance profile; b) storage modulus over 10 min, during which UV light was turned on 1 min after the test was started; c) Young's modulus; d) swelling ratio over 36 h; e) surface morphology at low and high magnifications, with dashed rectangles in the top row indicating the regions of magnified view shown in the corresponding bottom images (scale bar: low, 50 µm; high, 20 µm); f) pore size; g) porosity; h) 5.0MX/GG hydrogel showed higher electrical conductivity than GG hydrogel, as indicated by the higher brightness of the LED light; and i) electrical conductivity of hydrogels with varying MX concentrations.

The effects of MXene incorporation on the GG hydrogel were extensively investigated. It was found that MXene addition increased photo‐absorbance of GG hydrogels in the wavelength range of 330–1,000 nm (**Figure** **2**a). The storage modulus and Young's modulus of MX/GG hydrogels decreased with increasing MXene concentrations (Figure 2b,c), while the added MXene did not significantly affect their mechanical fatigue during a 10‐cycle compression testing (Figure S3, Supporting Information). An increase in MXene content was found to enhance the swelling ratio of GG hydrogels, with the 5.0MX/GG hydrogel showing the highest water absorption capability (Figure 2d). All MX/GG hydrogels exhibited porous structures, with their pore size and porosity both increasing at higher MXene loadings (Figure 2e–g). The addition of MXene accelerated the degradation of the GG hydrogels (Figure S4, Supporting Information). Notably, the incorporation of MXene, a 2D nanomaterial with excellent electrical conductivity,^[^ ^41^, ^42^ ^]^ into GG hydrogel resulted in significantly improved electrical conductivity, as shown in Figure 2h,i. These results collectively proved that the MX/GG hydrogels possessed satisfactory photo‐absorbance, high porosity, and excellent electrical conductivity.

### 2.2 Printability

**Figure 3:**
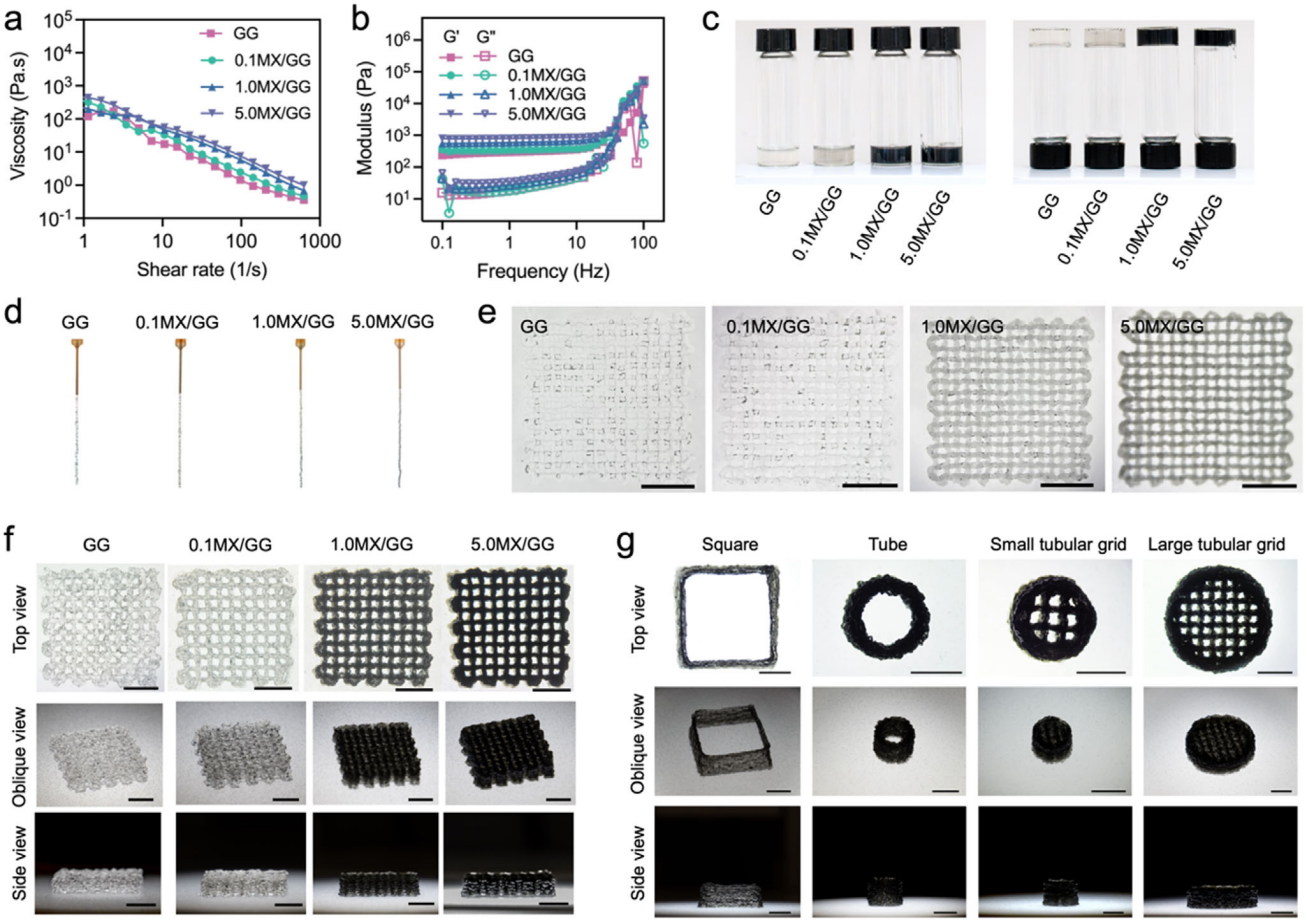
Printability of MX/GG inks. a) Shear viscosity of GG and MX/GG inks as a function of shear rate; b) loss modulus and storage modulus of the inks as a function of frequency; c) absence of gravity‐induced flow in inverted tubes containing inks, indicating gel‐like states; d) extruded filaments of different inks; e) pictures of one‐layer grids 3D printed from different inks; f) pictures of multi‐layer grids 3D printed from different inks, and g) pictures of different 3D structures fabricated by 3D printing. Scale bar: 5 mm).

The printability of MX/GG inks was evaluated in terms of their rheology and shape fidelity. The MX/GG inks demonstrated decreased viscosity with increasing shear rates (**Figure** **3**a), indicative of shear‐thinning behavior. This characteristic is essential for ensuring smooth flow through a nozzle, a crucial factor for successful extrusion‐based 3D printing. Figure 3b presents the inks’ storage modulus and loss modulus, revealing that MX/GG inks displayed higher storage modulus than loss modulus, signifying a gel‐like state. Consistent with this, the MX/GG inks maintained their stability when inverted for ≈10 min (Figure 3c). These results indicate that MX/GG inks possessed excellent shear viscosity and gel‐like properties, making them appropriate for extrusion‐based 3D printing. To further validate the printability of MX/GG inks, they were extruded using a 3D printer. Figure 3d demonstrates that MX/GG inks could be extruded as fine filaments after passing through a nozzle. These inks were also printed into a single‐layer grid with dimensions of ≈15 mm × 15 mm (Figure 3e). Additionally, multi‐layer grids fabricated from MX/GG hydrogels retained fine internal structures and clear pores post‐printing (Figure 3f), indicating excellent stackability of the MX/GG hydrogels. These multi‐layer grids were freeze‐dried and observed under SEM, showing good fidelity with regular macropore arrangement (Figure S5, Supporting Information). Besides grids, the 1.0MX/GG ink was 3D printed into other geometries, including stacked squares, tube, small tubular grid and large tubular grid (Figure 3g). These 3D‐printed hollow structures maintained good shape fidelity, indicating the self‐supporting capability of the MX/GG inks.

### 2.3 Shape Morphing Behavior

**Figure 4:**
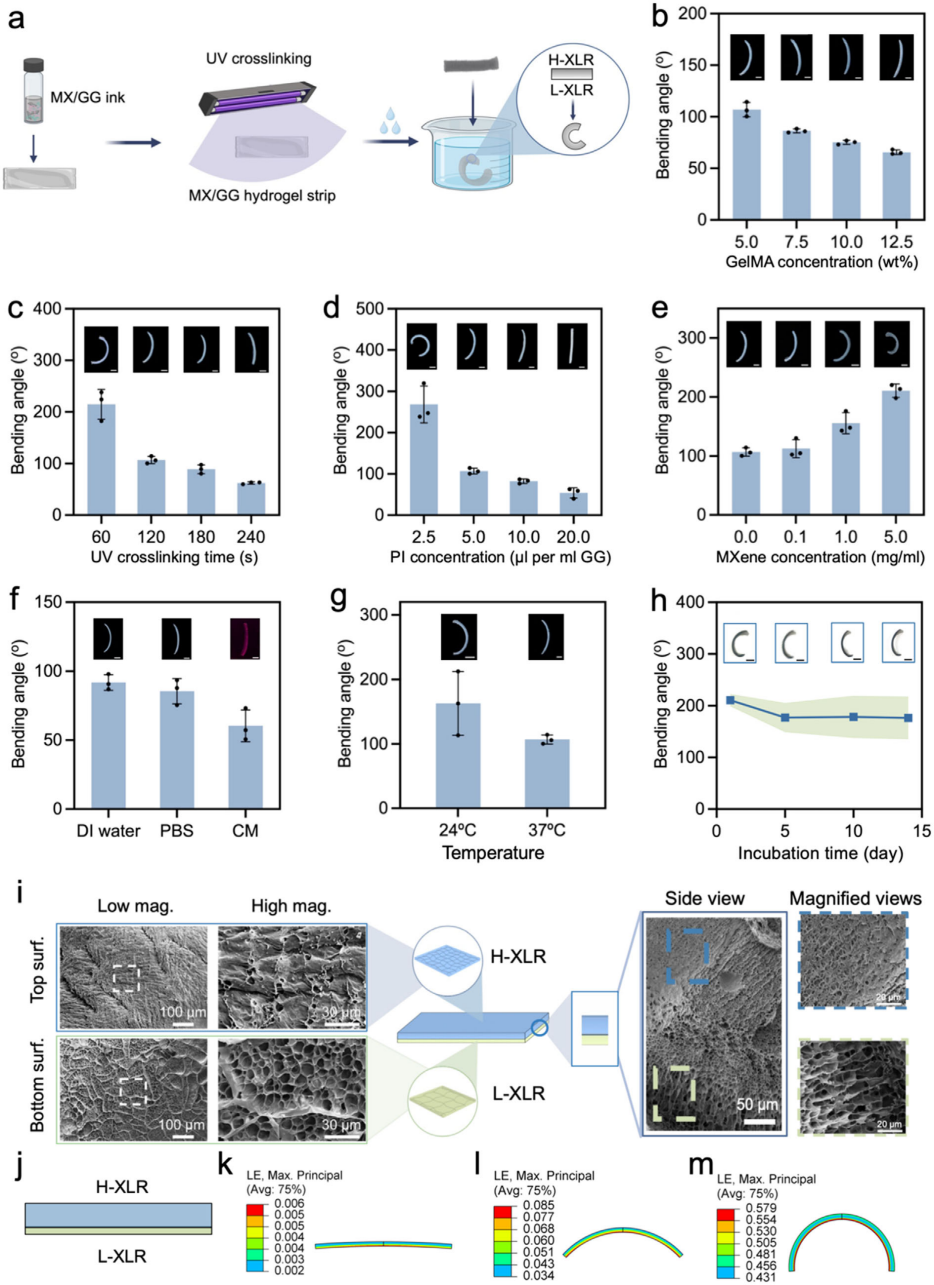
Optimization of the shape‐morphing behaviors of MX/GG hydrogels and elucidation of the underlying mechanism. a) Schematic illustration of the MX/GG hydrogel strips with self‐bending ability; b–g) Effects of different fabrication parameters and post‐fabrication treatments on the bending angle of MX/GG hydrogel strips, including (b) GelMA concentration, (c) UV crosslinking time, (d) photoinitiator (PI) concentration, (e) MXene content, (f) type of aqueous solutions, and (g) temperature of DI water used to soak the hydrogel (scale bar of the insets: 2 mm); h) bending angle of 5.0MX/GG strip incubated at room temperature for up to 14 days (scale bar of the insets: 2 mm); i) SEM images of the top surface, bottom surface, and cross section of freeze‐dried MX/GG hydrogels, with the dashed rectangles in the low‐magnification images (scale bar: 100 µm or 50 µm) indicating the areas of magnified view in the high‐magnification images (scale bar: 30 µm); and j) FEA modeling of MX/GG hydrogels with both H‐XLR and L‐XLR using a bilayer model and k–m) corresponding simulated bending results. The insets in (b–h) are photos of representative self‐deformed strips under the indicated conditions.

Shape morphing capability is vital for achieving a programmable 4D effect. For this test, MX/GG hydrogel strips with a dimension of ≈15 mm × 3 mm × 0.5 mm were created, as illustrated in **Figure** **4**a. Under precisely defined UV crosslinking conditions, a crosslinking gradient can be established across the thickness of the hydrogel strip.^[^ ^15^, ^16^, ^17^ ^]^ This crosslinking gradient resulted in uneven swelling and thus internal stresses, leading the hydrogel strip to self‐bend upon immersion in water. The bending angle of MX/GG hydrogel strips was affected by various parameters. Increasing the GelMA concentration led to a reduction in the curvature of the GG hydrogel strips (Figure 4b). Similarly, extending the UV crosslinking time and increasing the photoinitiator concentration both resulted in a decreased bending angle of the GG hydrogel strips (Figure 4c,d). Notably, the addition of MXene enhanced the folding of the GG hydrogel strips (Figure 4e). Due to its excellent UV absorption properties, MXene could act as a photoabsorber during UV crosslinking and promote the formation of a higher crosslinking gradient in the hydrogel strips,^[^ ^7^, ^8^ ^]^ thereby improving the shape‐morphing ability of the GG hydrogel. The type of aqueous solution used to trigger shape evolution also affected the bending of the GG hydrogel strips, with cell culture medium resulting in a lower curvature than DI water or PBS (Figure 4f). An incubation temperature of 37 °C was found to result in a decreased curvature than 24 °C (Figure 4g). Notably, the 5.0MX/GG hydrogel strip maintained well its bending angle during a two‐week incubation at room temperature (Figure 4h).

SEM was conducted to verify the crosslinking gradient of MX/GG hydrogel strips formed during UV crosslinking (Figure 4i). The top surface of the MX/GG hydrogel strips, which was closer to the UV light, exhibited a denser network with lower porosity compared to the bottom surface, which had a more porous network. The side view revealed the formation of the H‐XLR and L‐XLR near the top and bottom of the MX/GG hydrogel strips, respectively, indicating the creation of a graded hydrogel. Additionally, the crosslinking gradient was verified by the compressive stress‐strain measurements (Figure S6, Supporting Information) and swelling ratios of H‐XLR and L‐XLR (Figure S7, Supporting Information). To understand the self‐folding capability of such graded hydrogels, crosslinked MX/GG hydrogel strips were modeled as a bilayer structure consisting of an H‐XLR layer and an L‐XLR layer (Figure 4j). FEA simulation demonstrated that the bilayer MX/GG hydrogel strip showed a self‐bending shape that agrees well with the experimental results (Figure 4k‐‐m; Video S1, Supporting Information). These experimental data and simulation results both confirmed the humidity‐triggered shape‐morphing behavior of MX/GG hydrogel strips due to the presence of a crosslinking gradient induced by a single, facile UV light exposure.

### 2.4 Thickness‐Modulated Curved Behavior

**Figure 5:**
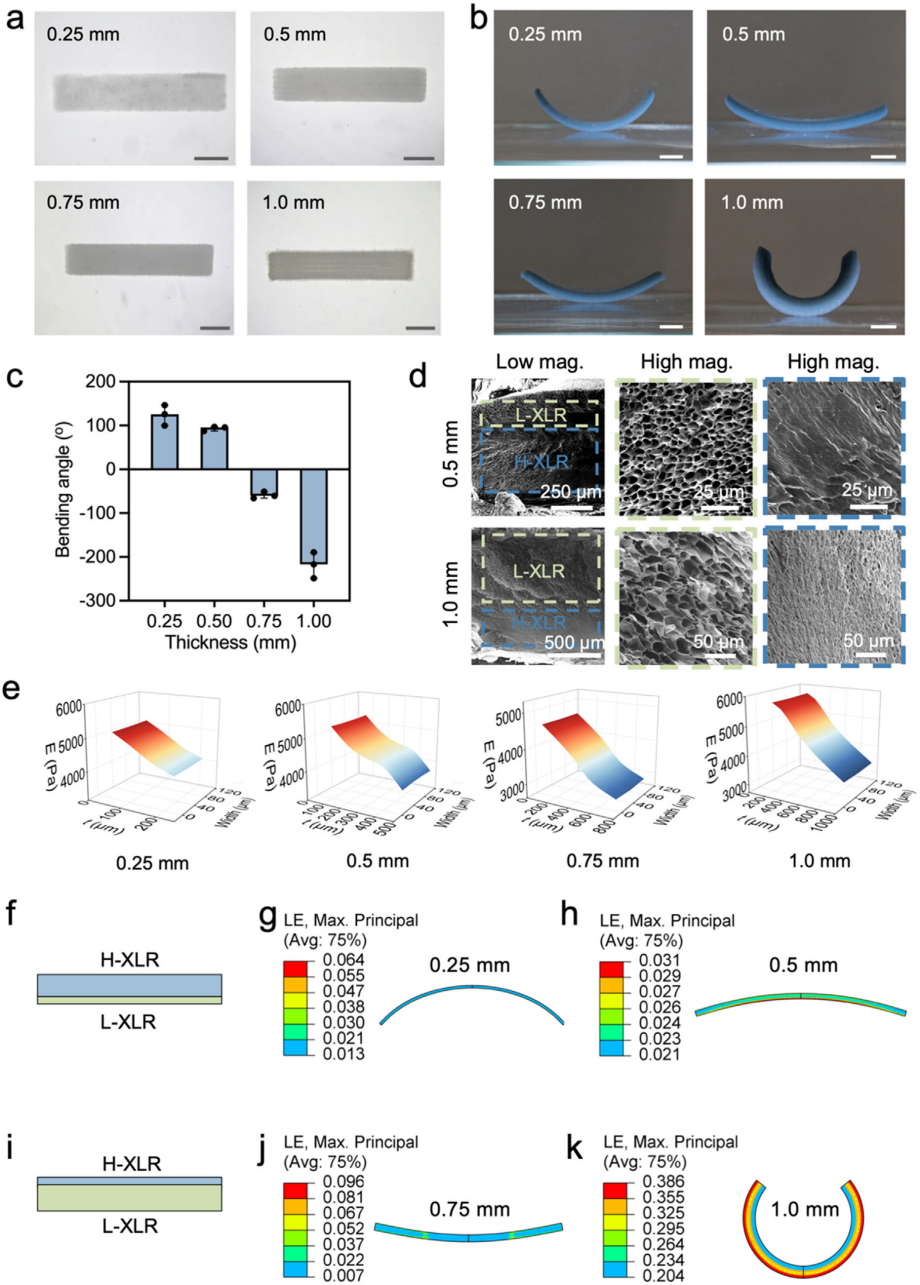
Thickness (t)‐dependent self‐folding behaviors of 4D printed 1.0MX/GG hydrogel strips and the underlying H‐XLR/L‐XLR ratio‐dependent shape‐morphing mechanism. a) Macroscopic images of 4D printed strips with different *t* values; b) macroscopic images of 4D printed strips with humidity‐triggered self‐bending (scale bar: 5 mm); c) quantification of the bending angle for hydrogel strips with different *t* values; d) SEM images showing the cross‐sectional view of 4D printed hydrogel strips with different *t* values; e) Cross‐sectional mapping of elastic modulus for hydrogel strips with different *t* values; f) a FEA bilayer model of hydrogel strips with *t≤*0.5 mm; g,h) simulated bending behaviors of strips with a *t* of (g) 0.25 mm and (h) 0.5 mm; i) a FEA bilayer model of hydrogel strips with *t≥*0.75 mm; j,k) simulated bending behaviors of strips with a *t* of (j) 0.75 mm and (k) 1.0 mm.

With excellent printability and shape morphing ability, MX/GG hydrogels are appealing candidates for 4D printing. To explore their potential as a 4D printing ink, 1.0MX/GG hydrogel was 4D printed into 20 mm × 4 mm strips with thickness values ranging from 0.25 to 1.0 mm (**Figure** **5**a). When immersed in DI water, these strips exhibited drastically different bending degrees (Figure 5b; Video S2, Supporting Information). Interestingly, the strips with a thickness of 0.25 mm and 0.5 mm self‐bent in a direction opposite to those with a thickness of 0.75 and 1.0 mm (Figure 5c). To understand the observed thickness‐dependent bending angle and direction, the cross‐sectional microstructure was examined using a fluorescent‐based method and SEM, and the mechanical properties of the swollen strips was evaluated using nanoindentation. The fluorescence image (Figure S8, Supporting Information) showed the fluorescence intensity gradient across the thickness direction, verifying the regions with crosslinking gradients generated in the hydrogels. Cross‐sectional SEM images (Figure 5d) and mechanical profiling (Figure 5e) confirmed gradients in microstructure, with the formation of H‐XLR and L‐XLR zones, and in elastic modulus. However, the relative areas of L‐XLR and H‐XLR varied greatly in hydrogel strips of different thicknesses. In thinner hydrogel strips (e.g., 0.25 and 0.5 mm thickness), the H‐XLR was more prominent than the L‐XLR. When the strip thickness was increased to 0.75 mm or higher, L‐XLR became dominant. Therefore, the direction of humidity‐triggered hydrogel bending relies on the relative proportions of H‐XLR and L‐XLR within the construct. For thin hydrogel strips dominated by H‐XLR, the high rigidity of H‐XLR possibly played a decisive role in hydrogel bending toward the relatively thin and soft L‐XLR. By contrast, in thick hydrogel strips, the swelling of dominant L‐XLR resulted in strip bending toward the H‐XLR. By modulating the ratio of L‐XLR to H‐XLR, which can be conveniently tuned, the hydrogels’ shape‐morphing behaviors can be effectively controlled.

To further verify this H‐XLR/L‐XLR ratio‐driven bending mechanism, ABAQUS FEA software was employed to simulate the self‐deformation of 4D‐printed hydrogel strips with different H‐XLR/L‐XLR ratios (Figure 5f,i). Consistent with the experimental findings (Figure S9, Supporting Information), the simulation results demonstrate that thin strips with thicknesses of 0.25 mm (Figure 5g) and 0.5 mm (Figure 5h) exhibited a different bending direction compared to those with thicknesses of 0.75 mm (Figure 5j) and 1.0 mm (Figure 5k). Collectively, these experimental and simulation results prove that different bending directions can be effectively achieved for hydrogel strips by simply adjusting their thickness under the same single‐dose UV exposure. This facile strategy offers an effective approach to constructing complex curved structures via 4D printing.

### 2.5 4D Printing of Complex Single‐ and Bidirectional Shape‐Morphing Constructs

**Figure 6:**
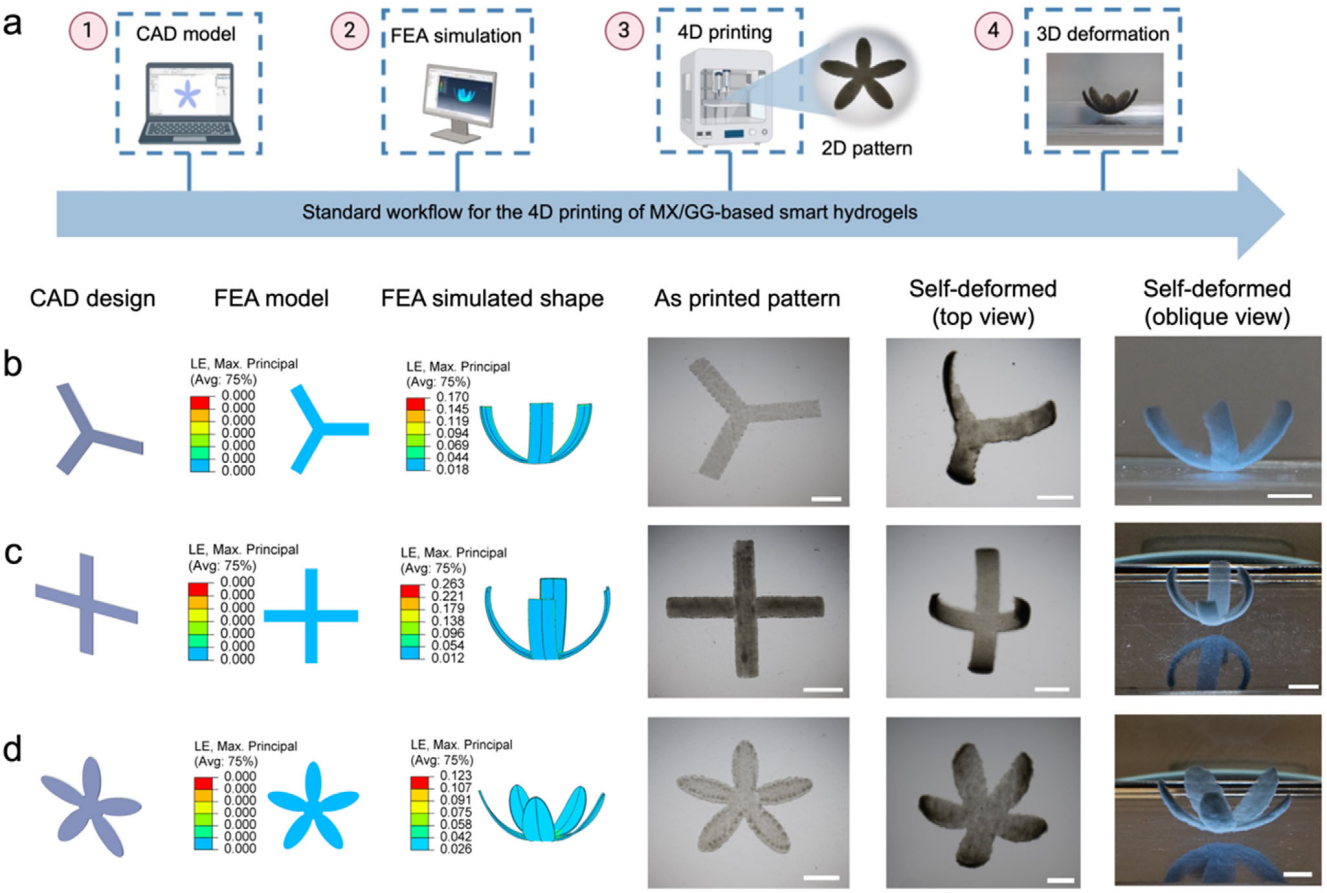
FEA simulation‐guided 4D printing of constructs with unidirectional self‐bending in response to humidity. a) Schematic illustration of the 4D printing process; b–d) different 4D printed unidirectional self‐bending constructs, illustrated with (from left to right) their CAD design, FEA model, simulated self‐bending behavior, and photographs of the as‐printed patterns and the self‐deformed 3D structure, including (b) three‐arm gripper, c) four‐arm gripper, and d) five‐petal flower. Scale bars: 5 mm.

The above experimental and FEA results have enabled us to establish a standard workflow for the 4D printing of MX/GG‐based smart hydrogels, as illustrated in **Figure** **6**a. First, a CAD model with defined dimensions is designed using a CAD software such as AutoCAD and SolidWorks. The designed CAD model is then imported into an FEA software and configured as a bilayer structure comprising H‐XLR and L‐XLR. Upon achieving an optimized design resulting in a desired 3D deformation in the FEA simulation, the CAD model is sliced for processing by an extrusion‐based 3D printer, which then fabricates the pattern of smart hydrogel. Finally, the 4D printed pattern is transformed to a programmed 3D morphology by responding to humidity.

To validate this 4D printing protocol, we first fabricated various complex constructs showing unidirectional bending by depositing planar patterns with a uniform thickness. Figure 6b illustrates a 3D three‐arm gripper derived from a planar pattern with three strips that were ≈0.25 mm thick. Additionally, a planar cross with four strips was designed, and FEA simulations indicated that these strips fold in the same direction to form a four‐arm gripper. The post‐printing deformation of the cross verified the simulation results (Figure 6c; Video S3, Supporting Information). Furthermore, a floral design with five petals, all 0.25 mm thick, was created and simulated using FEA. FEA simulation predicted the transformation of this planar pattern into a 3D five‐petal flower, which was again confirmed by our experimental observation (Figure 6d; Video S4, Supporting Information). The good agreement between the FEA simulation and actual deformation of 4D printed constructs supports the robustness and reliability of the established 4D printing protocol.

**Figure 7:**
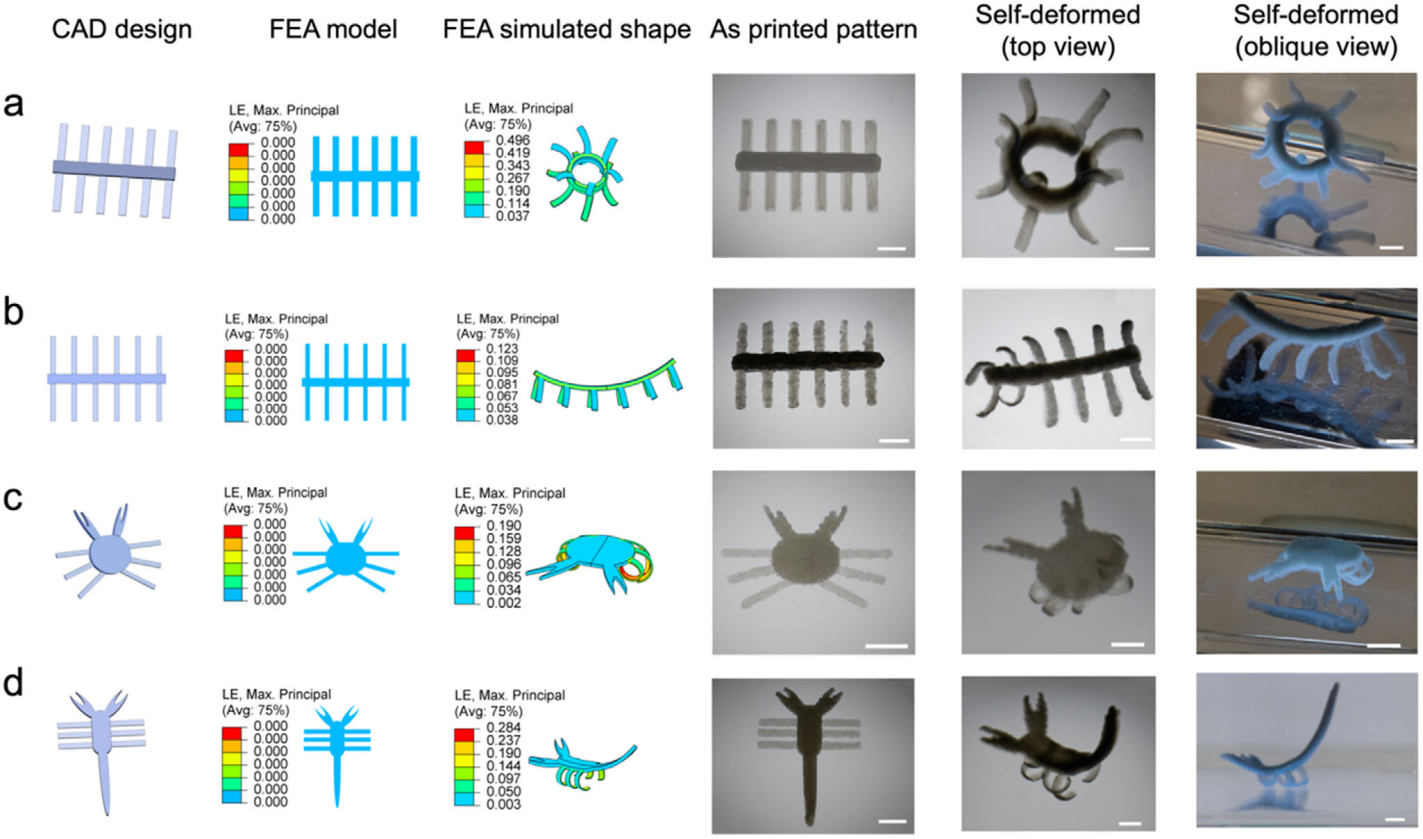
FEA simulation‐guided 4D printing of complex constructs with bidirectional self‐bending in response to humidity. a–d) Different biomimicking constructs illustrated (from left to right) with their CAD model design, FEA‐simulated deformation, and photographs of the as‐printed pattern and self‐deformed 3D constructs: (a) a coral polyp‐like structure with a circular body and its feet bending outward; (b) a millipede‐like structure with its body bending upward and feet bending downward; (c) a crab‐like structure with minimal bending of its body and claws but its feet folding downward; (d) a scorpion‐like structure with its pedipalps and tail bending upward and feet folding downward. Scale bar: 5 mm.

Native tissues often exhibit complex shape‐morphing capabilities characterized by multi‐directional curvatures. To demonstrate the versatility of this 4D printing strategy, a series of intricate constructs corresponding to the shapes of natural living organisms were printed by encoding multiple shape‐morphing domains. Distinct network gradients could be generated in shape‐morphing domains with different thickness under a single UV exposure. For example, based on FEA‐simulated shape changes, a pattern was printed featuring a 1 mm thick body strip and 0.25 mm thick feet strips. When swelling in DI water, this pattern transformed into an intricate 3D form, with the body forming a ring and the feet extending outward, akin to a coral polyp structure (**Figure** **7**a). Additionally, FEA results predicted that by reducing the body thickness and feet width of this pattern, the deformed construct could resemble a millipede. This was verified in our 4D printing experiments, in which the millipede's body slightly arched upward and its feet curved downward (Figure 7b; Video S5, Supporting Information). Using the same strategy, we successfully 4D printed lifelike constructs resembling a crab (Figure 7c) and a scorpion (Figure 7d). In both cases, FEA simulation reliably predicted the resultant curvature. These findings demonstrate the effectiveness of this 4D printing strategy in programming soft biomaterials into complex, dynamic architectures showing bidirectional self‐bending upon water immersion, which has been challenging to achieve in conventional manufacturing processes.

### 2.6 4D Bioprinting of Cell‐Laden and Self‐Folding Constructs

**Figure 8:**
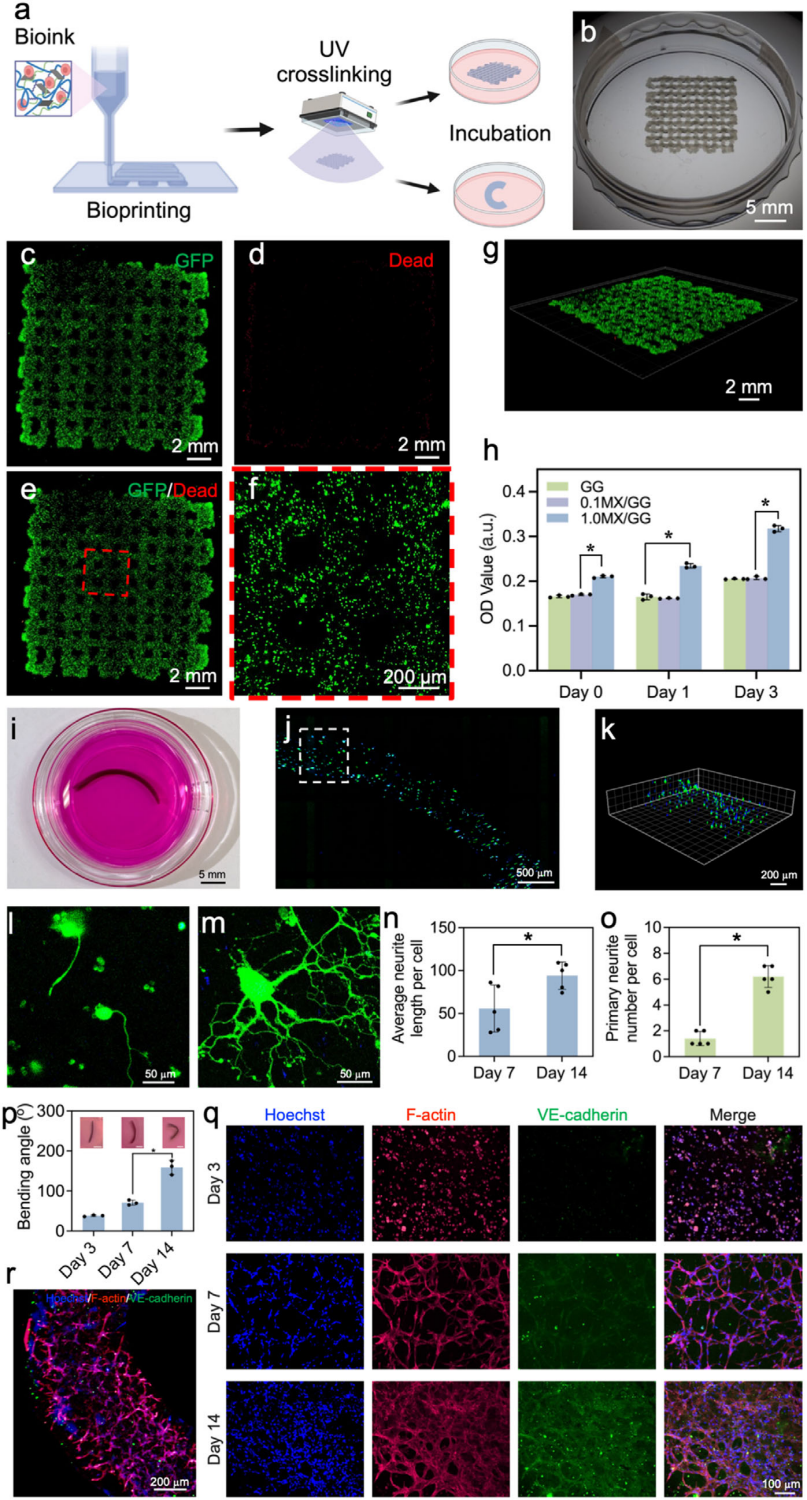
4D bioprinting of cell‐laden MX/GG hydrogels. a) Schematic of the 3D bioprinting process for two differently shaped constructs. b–g) Bioprinted grid of PC12 cell‐laden 1.0MX/GG hydrogel supported high cell viability and proliferation: (b) macroscopic image of the bioprinted grid, (c‐f) fluorescence images showing the (c) live cells in green, (d) dead cells in red, (e) merged image, (f) a magnified view of the dashed square in (e), (g) oblique view of the bioprinted grid. h) Viability of embedded PC12 cells as detected by CCK‐8 assay in MX/GG hydrogels after 0, 1, and 3 days of culture. i–o) 4D bioprinted self‐bending, PC12 cell‐laden constructs: (i) a 4D bioprinted strip of PC12 cell‐laden 1.0MX/GG hydrogel capable of self‐folding in culture medium, (j) fluorescence image presenting cell distribution in cross‐sectional view of the self‐bending strip, (k) a high‐magnification oblique view of the dashed rectangle in (j) showing PC12 cells within the strip, (l,m) fluorescence images showing PC12 cell differentiation in the self‐bending strips after (l) 7‐day and (m) 14‐day in vitro culture, and corresponding (n) average neurite length and (o) primary neurite number. p–r) 4D bioprinted self‐bending HUVEC‐laden constructs: (p) bending angle of the HUVEC‐laden strips during 14 days of in vitro culture, (q) fluorescence images showing the changes in HUVEC density and morphology in the hydrogel strips over 14 days, and (r) confocal image of the self‐bending strip after 14‐day in vitro incubation.

Finally, the potential of 4D printable MX/GG hydrogels in fabricating programmable, dynamic living scaffolds was explored. Prior to loading living cells into the hydrogels, the potential cytotoxicity of MXene at concentrations ranging from 0 to 5 mg/ml was evaluated (Figure S10, Supporting Information). It was found that MXene did not affect the cell viability, proliferation, or cell‐hydrogel interaction in vitro, demonstrating the excellent biocompatibility and cytotoxicity of MXene. After that, the cytocompatibility of MX/GG hydrogels was evaluated with two cell lines – 1) PC12 cells, an established neuronal cell model widely used in toxicology research; and 2) HUVEC, an epithelial cell line. For PC12 cells, as shown in Figure S11 (Supporting Information), Live/Dead staining images revealed significantly higher cell viability for cultures in 1.0MX/GG hydrogels than GG and 0.1MX/GG hydrogels. This difference could be attributed to the varying UV shielding capabilities of the different hydrogels. Compared to GG and 0.1MX/GG hydrogels, which have been shown to inadequately protect cells from cytotoxic UV irradiation during photocrosslinking,^[^ ^47^ ^]^ the 1.0MX/GG hydrogels demonstrated enhanced cytoprotection and reduced photodamage during crosslinking, likely due to MXene's high UV absorption capacity.^[^ ^48^, ^49^ ^]^ Given its excellent cytocompatibility, the 1.0MX/GG ink laden with living cells was selected to evaluate its bioprintability. **Figure** **8**a illustrates the bioprinting process of the cell‐laden 1.0MX/GG hydrogels. First, the PC‐12‐laden bioink was 3D bioprinted into grids with good shape fidelity and fine internal pore structure (Figure 8b). Live/Dead staining images (Figure 8c–g) confirmed uniform cell distribution and high viability within the constructs, indicating minimal cellular damage resulting from shear stress during extrusion or UV exposure. Furthermore, CCK‐8 assay results corroborated the high viability of PC12 cells in 1.0MX/GG hydrogels (Figure 8h).

Leveraging the excellent shape‐morphing properties of 1.0MX/GG hydrogels, we further used this composite ink to 4D bioprint living constructs. 4D bioprinted strips containing PC12 cells exhibited self‐bending behaviors and transformed into 3D curved architectures upon swelling in the culture medium (Figure 8i). PC12 cells were homogenously distributed in the 3D curved construct (Figure 8j,k). Following 7 and 14 days of differentiation, PC12 cells displayed progressive neurite outgrowth, marked by significant increases in both neurite length and branching density (Figure 8l–o). These findings demonstrate that 1.0MX/GG hydrogels possessed excellent 4D bioprintability and effectively supported the neuronal differentiation of PC‐12 cells, indicating their potential in creating programmable self‐folding neural constructs.

We also utilized another cell model, i.e., HUVECs, because their self‐organizing ability is particularly intriguing in the 4D bioprinting of dynamic tissues. During a 14‐day culture period, the HUVEC‐laden 1.0MX/GG hydrogels experienced increasing curvature (Figure 8p), which likely arose from time‐dependent cell traction forces generated by the proliferating and remodeling HUVECs. Immunofluorescence images of the embedded HUVECs revealed dynamic phenotypic transitions. At day 3, the cells displayed a rounded morphology with minimal VE‐cadherin expression, whereas by days 7 and 14, the cells exhibited pronounced spreading, upregulated VE‐cadherin expression, and self‐organized vascular network‐like structures (Figure 8q,r; Figure S12, Supporting Information). The evolving curvature was thus likely driven by increasing cytoskeletal tension and actin polymerization‐induced traction forces.^[^ ^50^, ^51^, ^52^, ^53^ ^]^ The emergence of endothelial networks within autonomously deforming scaffolds highlights the potential of MX/GG hydrogels in recapitulating vascular tissue morphogenesis, strongly suggesting that our 4D bioprinting strategy may serve as a promising technique for creating spatially programmable vasculature.

## 3 Discussion

4D bioprinting has garnered significant attention in recent years due to its capability to create dynamic tissues with programmable shape and/or functional evolution.^[^ ^1^, ^54^ ^]^ As this advanced technique is still at its initial stage, there is an urgent need to develop new 4D bioprintable smart hydrogels, along with facile and robust strategies for endowing these hydrogels with desirable 4D effects.^[^ ^27^ ^]^ This study addressed these needs by introducing a multifunctional bioink comprising MXene, gelatin and GelMA, and a facile smart design strategy based on thickness modulation. The hydrogel composites combined the photocrosslinkability of GelMA, rheological tunability of gelatin, and UV‐absorbing (shielding) and conductive properties of MXene, exhibiting excellent cytocompatibility, printability, and programmable shape morphing ability. The bioink could be fabricated into 4D constructs with bidirectional bending through a single‐step, UV‐mediated crosslinking gradient. Unlike prior strategies requiring multi‐material systems or toxic post‐printing processing,^[^ ^5^, ^24^, ^55^, ^56^, ^57^, ^58^ ^]^ our approach simplified complex shape programming into an FEA‐guided workflow centered at thickness and crosslinking gradient modulation at different shape domains. With the preservation of high cell viability during UV crosslinking and subsequent culture, the 4D bioprinted constructs showed both structural complexity and biological functionality, holding great promise in creating dynamic living tissues that recapitulate native morphogenesis and tissue functionalities.

Current 4D printing techniques, especially 4D bioprinting methods, rely heavily on soft natural hydrogels like GelMA, silk fibroin, and alginate derivatives,^[^ ^5^, ^15^, ^18^, ^57^ ^]^ which suffer from limited shape fidelity and/or simple deformation. Previously, Lewis’ group achieved complex humidity‐driven 4D biomimetic morphologies using composite hydrogels (comprising mainly cellulose nanofiber, nanoclay, and N,N‐dimethylacrylamide).^[^ ^59^ ^]^ However, their strategy excluded living cells, and thus its feasibility and scalability for 4D bioprinting needs to be verified. In another study, Ge's group employed bilayer dehydration for ceramic‐hydrogel hybrids to achieve bidirectional 3D deformation.^[^ ^55^ ^]^ However, their method also excluded living cells and involved non‐scalable processes that needed a high temperature and precise alignment via multi‐material bioprinting systems. Besides, previous gradient‐based printing methods,^[^ ^7^, ^8^ ^]^ while offering fine material‐level control, often necessitated the use of photomasks or other additional steps to achieve multi‐directional curvatures, which complicates the fabrication processes and limits its scalability. The MX/GG hydrogel developed in this study overcame these limitations by leveraging MXene's UV absorption, enabling spatially controlled crosslinking gradients. Applicable in a single‐step procedure, it obviated the need for multi‐material deposition and complex fabrication steps, and greatly improved the viability of encapsulated cells. The bidirectional deformation, achieved by varying the thickness to control the photocrosslinking gradient in specific areas, differs from the unidirectional bending observed in most previous studies.^[^ ^9^, ^15^, ^51^, ^60^ ^]^ The mechanism underlying the thickness‐dependent curvature is the H‐XLR/L‐XLR ratio within the hydrogels. Besides thickness, other factors can also affect the H‐XLR/L‐XLR ratio, such as UV intensity and crosslinking time. However, using these factors to control the L‐XLR/H‐XLR ratio at different shape‐morphing domains requires the use of photo masks or additional fabrication steps, which complicates the pattern design and 4D printing processes. Compared to existing 4D bioprinting strategies (Table S1, Supporting Information), the straightforward thickness‐controlled 4D bioprinting strategy, requiring only a single UV exposure, has the advantages of being easy to operate, a facile fabrication process, excellent shape morphing ability (supporting both unidirectional and bidirectional curvatures), and high cell viability. This underscores its scalability for creating biomimetic architectures (e.g., flower, millipede, and scorpion) without complicated fabrication steps.

The simplicity of achieving bidirectional bending through thickness‐controlled crosslinking gradients is remarkable. Prior studies mostly utilized complex computational models^[^ ^59^ ^]^ or multi‐material interfaces^[^ ^57^, ^58^, ^61^ ^]^ as prerequisites for multi‐axis deformation. Herein, the thickness‐dependent swelling anisotropy of MX/GG hydrogels enabled the production of predictable complex curvature via single‐nanocomposite 4D printing and single post‐printing UV exposure, potentially leading to a paradigm shift in 4D printing design philosophy. This greatly reduces the field's reliance on complex engineering solutions and underscores the underexplored potential of nanomaterial‐enhanced hydrogels for spatial stress modulation. Another noteworthy point was the multifunctionality of MXene in the hybrid ink. Numerous GG‐based bioinks have been reported for 3D bioprinting applications,^[^ ^29^, ^30^ ^]^ but their applications in 4D bioprinting are still limited due to the poor shape morphing capability. The introduction of MXene endowed GG hydrogel with electrical conductivity, UV shielding effect, and a larger crosslinking gradient for improved shape morphing capability. Within HUVEC‐laden constructs, endothelial self‐organization occurred and possibly generated internal mechanical stress that in return contributed to the increasing curvature of the strips over time.^[^ ^6^, ^51^, ^62^ ^]^ This finding highlights the hydrogel's ability to synergize swelling‐ and cell‐induced mechanical stresses.

While promising, the 4D printing strategy developed in this study has several limitations. First, the need for water to induce shape morphing of MX/GG hydrogels limits the applicability of the 4D printing strategy to aqueous environments. Thus, further technological modifications to achieve responsiveness to multi‐stimuli (e.g., temperature, pH) could broaden the utility of this strategy. Then, achieving complex curvature relies on thickness modulation and/or adjustment of photocrosslinking intensity, thus necessitating high printing resolution. The resolution of extrusion‐based 3D printing is constrained by the nozzle size (varying from several hundred µm to several mm), which physically limits this printing technique to the creation of millimeter‐scale shape morphing constructs and faces challenges in achieving shape transformation with higher precision. Next, the integration of living cells complicates the smart design for 4D bioprinting. While we have demonstrated the feasibility of creating PC12‐ and HUVEC‐laden, shape‐morphing strips, it warrants further exploration to generate more complex, dynamic cell‐laden 3D constructs. Additionally, the UV light used to crosslink hydrogels reduces cell viability. In the future, non‐UV light sources that are more cell‐friendly can be used to minimize cell damage during 4D bioprinting.^[^ ^63^, ^64^ ^]^

With notable simplicity and feasibility, our 4D bioprinting approach holds promising potential to be upscaled to fabricate clinically relevant tissue or organ constructs which require specific curvatures for matching the diseased tissues. Specifically, based on the computer tomography or magnetic resonance imaging data of a specific patient, the 3D curved model can be built and designed into specific patterns with the guidance of FEA. After that, the designed pattern is 4D bioprinted and deployed into the defect site for shape change to seamlessly match the defect site. This may necessitate advancements in bioprinting technology, such as the development of higher‐resolution printers, alongside comprehensive biological analysis of MXene‐containing bioinks. Our preliminary data indicates the high cytocompatibility of MXene with PC12 cells and HUVECs, suggesting its potential biological safety in vivo. However, its long‐term effects in vivo (e.g., toxicity, immune response, and degradation) remain to be explored. The bioinks are composed of gelatin, GelMA, and MXene. As GelMA and gelatin are both widely recognized for their high biocompatibility and low immunogenicity, we expect these constituents to elicit a minimal immune response, consistent with prior studies on similar biomaterials.^[^ ^65^, ^66^, ^67^ ^]^ MXene, as an emerging 2D nanomaterial, has also shown promising in vivo biocompatibility in recent research on different tissue regeneration applications, such as skin wound repair,^[^ ^68^, ^69^ ^]^ spine cord regeneration,^[^ ^70^ ^]^ supporting the biosafety of MXene‐containing bioink. In terms of tissue integration, the porous architecture and shape‐morphing properties of these MX/GG constructs are designed to promote cell attachment and growth and conform to the geometries of tissue defects, thereby likely enhancing integration with host tissues. Regarding construct degradation, gelatin and GelMA naturally degrade over time, which is desirable for tissue regeneration. Given that MXene's inclusion may alter the degradation profile of GG hydrogels, long‐term in vitro experiments and in vivo tests will be necessary in the future to fully understand the degradability of the nanocomposite ink.

In terms of costs, this 4D bioprinting approach applies a single smart nanocomposite hydrogel (MX/GG) and a single UV exposure step, which effectively reduces material and labour costs. Gelatin is highly affordable, and GelMA synthesis from gelatin induces low costs too. Although MXene is more expensive, its use at a very low concentration of 1 mg/ml minimizes its impact on the overall costs. While the prices of commercial bioprinting machines vary greatly, ranging from several thousands to tens of thousands of US dollars. Our simple 4D printing approach requires only a common extrusion‐based model, which costs a few thousand US dollars, making our printing strategy practical for research and small‐scale use. Overall, utilizing cost‐effective materials and affordable equipment, our 4D bioprinting strategy offers a highly feasible solution to potential tissue engineering challenges.

In summary, we report here an MXene‐enhanced hydrogel platform that exhibited structural programmability and biological functionality as a smart ink to advance 4D bioprinting. FEA was performed to successfully model swelling‐induced deformation and predict the shape morphing process from CAD designs. Our results have established a straightforward thickness‐modulated, FEA‐guided 4D printing protocol capable of programming complex curvatures using a single ink, bypassing the need for complex multi‐material systems. This workflow streamlined the fabrication of biomimetic architectures, ranging from single‐directional self‐folding constructs to those with bidirectional deformation. Additionally, with the ability to provide encapsulated cells with instructive mechanical signals during dynamic structural evolution, the MX/GG hydrogels not only act as passive scaffolds, but also actively participate in tissue morphogenesis. These advances address long‐standing challenges in smart bioink design and greatly expand the avenue for engineering dynamic tissues via 4D bioprinting.

## 4 Conclusion

We report here the facile 4D bioprinting of smart composite MX/GG hydrogels with high printability and shape‐morphing capability. The incorporation of MXene into the MX/GG hydrogels not only facilitated the formation of crosslinking gradients, but also enhanced the scaffolds’ electrical conductivity and protected living cells from UV during crosslinking. By controlling the thickness of 4D printed strips, different post‐swelling curvature directions can be achieved. This facile approach was verified by FEA simulation, which in turn can be applied to guide the design of desired 4D shape‐morphing effects. The FEA‐guided thickness‐controlled strategy allowed us to 4D print various biomimetic patterns, including single‐directional curved 3D morphologies like a five‐petal flower, and bidirectional curved 3D morphologies such as coral polyp and scorpion. Furthermore, cell‐laden self‐bending strips were successfully 4D bioprinted, with the embedded living cells demonstrating high growth and functionality during in vitro culture. In summary, the MX/GG smart hydrogels and versatile 4D printing strategy reported herein pave the way for innovative 4D bioprinting applications, enabling the creation of advanced programmable, shape‐morphing living constructs for regenerative medicine applications and beyond.

## 5 Experimental Section

### Materials

Gelatin (type A, from porcine skin) and methacrylic anhydride (MA) were purchased from Sigma–Aldrich (USA). Ti_3_C_2_ MXene was obtained from Xinxi Technology Co., Ltd., China. Gibco^TM^ phosphate buffered saline (PBS) was purchased from ThermoFisher Scientific Inc. (USA). GelMA was synthesized according to a previously established protocol.^[^ ^71^ ^]^ Briefly, 10% (w/v) gelatin was first dissolved in PBS at 60 °C. MA (60% of gelatin weight) was added to the gelatin solution. The reaction lasted ≈1 hour, and the mixture was transferred into dialysis tubing. The dialysis was performed against deionized water at 40 °C for one week, after which the GelMA solution was freeze‐dried over 5 days. The synthesized GelMA was characterized using ^1^H NMR (Ascend^TM^ 500, Bruker Corporation, USA) and the degree of modification was calculated according to the ^1^H NMR result (more details are in the supplementary file).

### Characterization of MX/GG Hydrogels—Preparation of MX/GG Hydrogels

The MX/GG composite hydrogels with different MXene concentrations ranging from 0 to 5 (mg mL^−1^) were prepared. First, a 10 mg mL^−1^ MXene solution was prepared by magnetically stirring MXene powder in DI water overnight. Afterward, the MXene solution was diluted to 0.1, 1, and 5 mg mL^−1^. Next, an MX/GG hydrogel composite was prepared by dissolving gelatin and GelMA, both at 5% (w/v), in MXene solution at 50°C. Finally, 2‐hydroxy‐2‐methylpropiophenone was added at 5 µL mL^−1^ as the photoinitiator into the MX/GG hydrogels and mixed thoroughly via magnetic stirring. The prepared MX/GG hydrogels with MXene concentrations of 0, 0.1, 1, and 5 mg mL^−1^ are termed as GG, 0.1MX/GG, 1.0MX/GG, and 5.0MX/GG, respectively.

### Characterization of MX/GG Hydrogels—Light Absorption Ability

The light absorption property of MX/GG composite hydrogels was analyzed using a microplate spectrophotometer (Multiskan GO, ThermoFisher Scientific Inc., USA) using a wavelength range of 330–1,000 nm.

### Characterization of MX/GG Hydrogels—Storage Modulus

The storage modulus of the MX/GG composite hydrogels was determined using a rheometer (Kinexus Lab+, Malvern Panalytical, UK). The uncrosslinked MX/GG precursors were placed between a 0.6 mm measurement gap. These hydrogel precursors were subjected to rheometric testing under a strain of 1% and a frequency of 1 Hz for a duration of 10 min. The radical polymerization of the MX/GG hydrogels was triggered by UV illumination 1 min after the testing was started.

### Characterization of MX/GG hydrogels—Young's Modulus

The MX/GG hydrogels were fabricated into disks with a diameter of ≈10 mm and a thickness of 5 mm via UV crosslinking. The specimens were immersed in PBS and their Young's modulus was determined using a nanoindenter (Optics11 Life, the Netherlands).

### Characterization of MX/GG hydrogels—Swelling Property

MX/GG hydrogels (*n* = 3 for each type of hydrogel) with different MXene concentrations were fabricated into disks and then air‐dried. The dry weight of each specimen was recorded as *W_d_*. The dried specimens were immersed in DI water for 36 h to allow for water absorption. At specific time points, the swollen specimens were taken out, and their swollen weight was recorded as *W_s_*. The swelling ratio of MX/GG hydrogels were calculated as *W_s_*/*W_d_*.

### Characterization of MX/GG hydrogels—Surface Microstructure

The surface microstructure of the MX/GG hydrogels was examined using a field emission scanning electron microscope (Hitachi SU8010 SEM, Japan). Briefly, the MX/GG hydrogels were frozen in liquid nitrogen and then freeze‐dried over 3 days. All freeze‐dried samples were then sputter‐coated with a thin layer of gold. The images of surface morphology were captured in the secondary electron imaging mode.

### Characterization of MX/GG hydrogels—Pore Size and Porosity

The pore size of MX/GG hydrogels was calculated using ImageJ software based on the SEM images. The porosity of these hydrogels were determined based on an ethanol‐exchange method.^[^ ^72^ ^]^ Briefly, the cast MX/GG hydrogel disks were freeze‐dried and their weight was recorded as *W_n_*. Next, the freeze‐dried samples were immersed in absolute ethanol and subjected to vacuum until the MX/GG hydrogel was full of ethanol. The weight of the ethanol‐infiltrated samples was recorded as *W_e_*. The porosity of the MX/GG hydrogels was calculated using the following formula:

(1) $$porosity=100\%\times \left(w_{e}-w_{n}\right)/\rho V$$

where *ρ* is the density of absolute ethanol and *V* is the volume of dried hydrogel.

### Characterization of MX/GG Hydrogels—Electrical Conductivity

MX/GG hydrogels were cast into disks with a diameter of ≈10 mm and a thickness of ≈5 mm. The size of each specimen was measured using a digital caliper. The electrical resistance was measured using a multimeter system. The electrical conductivity (*σ*) of MX/GG hydrogel disks was calculated using the following formula:^[^ ^73^ ^]^

(2) σ=L/RA

where *L* is the thickness, *A* is the cross‐sectional area, and *R* is the electrical resistance.

### Printability—Rheology

The rheological properties of MX/GG inks were analyzed using a rotational rheometer (Kinexus Lab+, Malvern Panalytical, UK). Two types of rheologic experiments were conducted: 1) shear thinning property; and 2) angular frequency sweep. The shear thinning property of MX/GG inks with different MXene contents was tested over a shear rate range of 1 to 1000 1/s with a 0.55 mm measurement gap. The angular frequency sweep was conducted to measure the storage modulus (G') and loss modulus (G'') in a frequency range of 1 to 100 rad s^−1^ at a constant strain of 1%. Additionally, tubes containing uncrosslinked MX/GG hydrogels were inverted for ≈10 min to examine the occurrence of possible gravity‐induced flow.

### Printability—Shape Fidelity of 3D Printed Constructs

To assess shape fidelity, MX/GG hydrogels were manufactured into different 3D structures using an extrusion‐based 3D bioplotter (EnvisionTEC, Germany). First, to investigate their extrudability, the state of extruded hydrogel filaments was carefully examined, and one‐layer grids were printed from different MX/GG inks. Next, multi‐layer grids were 3D printed to evaluate the stackability of MX/GG hydrogels. Finally, different more complex 3D structures such as tubular and columnar structures were fabricated to demonstrate the 3D printability of MX/GG hydrogels.

### Shape Morphing Behavior

To investigate the hydrogels’ shape morphing behaviors under different conditions, MX/GG hydrogel precursors were fabricated into strips with a dimension of 15 mm × 3 mm × 0.5 mm under UV light, with a wavelength of 365 nm and a power of 20W, placed 5 cm above the sample (UV LED lamp, ZLUV LAMP, China). The hydrogel strips were then immersed in DI water for ≈1 h to reach maximal bending. The bending strip was imaged using a digital single lens mirrorless (DSLM) camera (A7m4, Sony, Japan). Also, the hydrogel strips were freeze‐dried, and their cross‐section view as well as top‐ and bottom‐surface morphology were observed under SEM. The cross‐sectioned mechanics of the hydrogel strips were analyzed using the nanoindenter.

### Finite Element Analysis

The fluid diffusion into MX/GG hydrogels and their shape deformation were simulated using a coupled diffusion‐deformation theory.^[^ ^74^, ^75^, ^76^ ^]^ This numeric simulation was implemented utilizing a user‐element subroutine (UEL) with a FEA software (ABAQUS). In the simulation, a coupled temperature‐displacement analysis was employed that utilized user elements with degrees of freedom. The 4D fabricated MX/GG hydrogel patterns were modeled as a bilayer structure, consisting of a high crosslinking region (H‐XLR) and a low crosslinking region (L‐XLR). The two layers were assumed to be perfectly bonded. The geometries of the 4D fabricated structures and their boundary conditions were consistent with the actual experimental setups (More details are in the supplementary file).

### FEA Simulation‐Guided 4D Printing

The same 3D bioplotter was used to 4D print MX/GG hydrogels with various simple and complex 2D patterns capable of programmable shape transformation. Initially, a virtual 2D pattern were designed using a CAD software (SolidWorks). The CAD design was then imported into ABAQUS FEA software to predict their shape morphing process. Upon achieving the desired shape transformation, the designed CAD model was sliced to generate the printing paths using a slicing software compatible with the 3D bioplotter. Subsequently, according to the sliced data, the MX/GG hydrogels were 4D printed and subjected to UV crosslinking. Finally, the 4D printed MX/GG pattern was immersed in DI water for ≈1h to undergo 3D deformation. The 3D morphology of the 4D printed structures was captured using the DSLM camera.

### Cells and Cell Culture

Two types of cell models were used in this study: PC 12 cells [supplied from American Type Culture Collection, USA; Research Resource Identifier (RRID): CVCL_0481; obtained on 20th March 2023] and HUVECs [obtained from Lonza, Switzerland; RRID: CVCL_2959; obtained on 13th September 2024]. PC12 cells are a widely used neuronal cell model commonly used for neuroscience and toxicology research. HUVECs are selected as another cell model because their self‐organizing ability is particularly intriguing in the biofabrication of dynamic tissues. PC 12 cells were cultured in RPMI 1640 culture medium (Gibco, ThermoFisher, USA) supplemented with 5% horse serum (ThermoFisher), 5% fetal bovine serum (FBS, Gibco), and 1% penicillin‐streptomycin (Gibco). HUVECs were cultured in high‐glucose Dulbecco's modified eagle medium (DMEM, Gibco) supplemented with 10% FBS, and 1% penicillin‐streptomycin. In vitro cell culture was performed in a humidified incubator at 37 °C with 5% CO_2_ and the culture medium was renewed every 2 d. When cells reached 80%–90% confluence, they were detached, counted, and subjected to preparing cell‐laden bioinks for the subsequent cytocompatibility evaluation and bioprinting. The cells were regularly tested to ensure that they were free from contamination.

### Cytocompatibility of Smart MX/GG Hydrogels

To evaluate the hydrogels’ cytocompatibility, PC12 cells were homogeneously mixed with filter‐sterilized MX/GG hydrogel precursors at a cell density of ≈2×10^6^ cells mL^−1^. Cell‐laden MX/GG hydrogel disks were fabricated via UV crosslinking and incubated in the RPMI 1640 culture medium. The viability of PC12 cells was assessed using a Live/Dead assay (ThermoFisher), and fluorescent images were captured using an EVOS M5000 Microscope (ThermoFisher), where live and dead cells showed green and red fluorescence, respectively. The proliferation of cells in the hydrogels was determined using a cell counting kit‐8 (CCK‐8) assay (Apexbio Technology LLC, USA).

### 4D Bioprinting of Living Constructs

Bioinks composed of gelatin, GelMA, MXene, and living cells were first prepared. In addition to PC12 cells, HUVECs as another cell model in the biofabrication of dynamic tissues were selected. The two types of cells were individually encapsulated within sterilized 1.0MX/GG solutions. Next, a pre‐sterilized 30 CC syringe was loaded with the bioink and mounted onto the 3D bioplotter. First, a cell‐laden grid was 3D bioprinted using the PC12‐laden bioink. Additionally, self‐folding strips were 4D bioprinted using bioinks containing PC12 cells or HUVECs. After bioprinting, the cell‐laden constructs underwent UV crosslinking using a UV LED lamp. The grids were crosslinked for 3 min, the PC‐12 laden strips for 1 min, and the HUVEC‐laden strips for 3 min. A shorter duration of 1 min was applied to achieve a relatively soft hydrogels for optimal PC12 cell differentiation, as neural cells are very sensitive to stiffness and stiff hydrogels may inhibit neurite outgrowth in 3D cultures.^[^ ^30^ ^]^ In contrast, a longer duration of 3 min was used to ensure structural stability in HUVCE‐laden constructs without affecting tube formation during in vitro culture. The UV intensity remained constant across all experiments to avoid variations in UV power when studying the effect of exposure time. To enable time‐dependent shape morphing and neuronal differentiation, the strips containing PC12 cells were incubated at the RPMI 1640 differentiation medium supplemented with 1% horse serum, 1% penicillin‐streptomycin, and 50 ng mL^−1^ nerve growth factor (Alomone Labs, Israel). After in vitro culture for 7 and 14 days, the differentiation of GFP‐transfected PC12 cells was assessed using a confocal laser scanning microscope (Leica SP8 STED, Germany). The HUVECs‐laden strips were incubated in the high‐glucose DMEM culture medium. On days 3, 7, and 14, the shape of the strips was captured using a digital camera. For phenotypic analysis, the HUVECs embedded in the strips were fixed and stained with Hoechst, F‐actin, VE‐cadherin. Briefly, HUVECs laden in the strips were first fixed with Formalin, permeabilized with 0.3% (v/v) Triton X‐100, and blocked using 1% (wt) bovine serum albumin. The embedded HUVECs were then labelled with VE‐cadherin monoclonal antibody (ThermoFisher) at a concentration of 5 µg mL^−1^, incubated at 4 °C overnight. Afterward, the cells were labelled with goat anti‐mouse secondary antibody (ThermoFisher) at a concentration of 5 µg mL^−1^. Finally, the cellular cytoskeleton was stained with F‐actin (Rhodamine Phalloidin, ThermoFisher) and nuclei were stained with Hoechst (ThermoFisher). The fluorescence imaging was performed on the confocal microscope (Leica MICA, Germany).

### Statistical Analysis

All quantitative data was presented as mean ± S.D. Statistical analysis was performed using one‐way analysis of variance using IBM SPSS Statistics software. A value of p < 0.5 represented statistical significance.

## Conflict of Interest

The authors declare no conflict of interest.

## Supporting information



Supporting Information

Supplemental Video 1

Supplemental Video 2

Supplemental Video 3

Supplemental Video 4

Supplemental Video 5

## Data Availability

The data that support the findings of this study are available from the corresponding author upon reasonable request.
