# Supplementary material for: Facile Single‐Nanocomposite 4D Bioprinting of Dynamic Hydrogel Constructs with Thickness‐Controlled Gradient
Source: Adv Sci (Weinh). 2025 Jul 14;12(39):e09449. doi: 10.1002/advs.202509449 (PMC12533157; doi:10.1002/advs.202509449)
Supplement: Supplementary file 1 — Supporting Information [file ADVS-12-e09449-s003.pdf]

ADVANCED SCIENCE
Open Access

Supporting Information

for *Adv. Sci.*, DOI 10.1002/advs.202509449

Facile Single-Nanocomposite 4D Bioprinting of Dynamic Hydrogel Constructs with Thickness-Controlled Gradient

Jiahui Lai, Tiandi Xiong, Shangsi Chen, Zhilong Zhou, Jun Liu, Boguang Yang, Rocky S. Tuan and Zhong Alan Li**

Supplementary Material

Facile Single-nanocomposite 4D Bioprinting of Dynamic Hydrogel Constructs with Thickness-Controlled Gradient

Jiahui Lai[1,2], Tiandi Xiong[1,2], Shangsi Chen[1,2], Zhilong Zhou[1], Jun Liu[1,2], Boguang Yang[1], Rocky S. Tuan[1,2,3]*, Zhong Alan Li[1,2,3,4]*

[1]Department of Biomedical Engineering, The Chinese University of Hong Kong, NT, Hong Kong SAR, P.R. China
[2]InnoHK Center for Neuromusculoskeletal Restorative Medicine, Hong Kong Science Park, NT, Hong Kong SAR, P.R. China
[3]Institute for Tissue Engineering and Regenerative Medicine, School of Biomedical Sciences, The Chinese University of Hong Kong, Hong Kong SAR, P.R. China
[4]Shun Hing institute of Advanced Engineering, The Chinese University of Hong Kong, Hong Kong SAR, P.R. China

*Correspondence: alanli@cuhk.edu.hk (Z.A.L.); tuanr@cuhk.edu.hk (R.S.T.).

^{1}H NMR characterization of synthesized GelMA

After GelMA was synthesized based on chemical reaction shown in Figure S1a, it was characterized using ^{1}H NMR (AscendTM 500, Bruker Corporation, USA). The synthesized GelMA and gelatin were separately dissolved in deuterium oxide (D_2O, procured from Shanghai Aladdin Biochemical Technology Co., Ltd., China) at 20 mg/mL. A 500 µl aliquot of each prepared solution was transferred into an NMR tube, which was subsequently analyzed using the ^{1}H NMR spectrometer to obtain their spectra. As shown in Fig. S1b, the GelMA spectrum displayed distinctive peaks indicated by a and c, which correspond to the proton signals of the methacrylate vinyl group present in methacrylic anhydride. Additionally, the intensity of peak b, which represents the proton signal of lysine methylene, was significantly reduced in GelMA, suggesting that the methacrylic group reacted with the lysine of unmodified gelatin to form GelMA. Given that the lysine group is a pivotal reactant in GelMA synthesis, the intensity of this peak in both GelMA and gelatin samples was used to calculate the degree of modification (DoM) of GelMA according to the following equation [1]:

$$DoM = \left(1 - \frac{A_{GelMA}}{A_{Gelatin}}\right) \times 100\% \qquad (1)$$

where $A_{gelatin}$ and A_{GelMA} represent the integrals of the lysine methylene signals of GelMA and gelatin, respectively.

According to Equation 1, the DoM of the synthesized GelMA was calculated to be ~50%.

To demonstrate the photocrosslinking ability of the synthesized GelMA, a 5 wt.% GelMA precursor was prepared. After UV crosslinking for 3 min, the GelMA precursor was solidified and remained stable after inverting the glass vial (Figure S1c).

Characterization of purchased MXene

The purchased $Ti_3C_2T_x$ MXene was characterized using different methods before use. Fig. S2a presents the photograph of MXene dispersed in DI water at different concentrations ranging from 0 to 5 mg/ml. MXene can be homogenously dispersed in DI water and the solution color become darker with the increasing MXene contents.

Fig. S2b shows the UV-vis-NIR absorption spectrum of MXene. As can be seen, MXene absorbs broad spectrum at a wavelength range of 330 to 1000 nm with an absorption peak around 800 nm [2]. The X-ray Diffraction (XRD) pattern (Fig. S2c) shows the characteristic (002) peak at 2θ=6.3°, indicating the substitution of the Al layers in Ti_3AlC_2 with the surface-terminating groups (T_x) and formation of $Ti_3C_2T_x$ MXene [3, 4]. Additionally, the Raman spectrum shown in Fig. S2d demonstrates the formation of titanium dioxide, as indicated by the presence of anatase (a) phase peak at 208.7 cm^{-1} and the rutile (r) peaks at 393.8 cm^{-1} and 581.1 cm^{-1} [5, 6]. Fig. S2e and S2f show the TEM images of MXene from the top view and side view, where typical exfoliated MXene flakes were observed. Fig. S2g shows the SEM image of MXene flakes where an opened interspace and the layered structure are obviously observed [5]. All these results confirm the available and good quality of the purchased MXene used in this study.

Hydrogel degradation

The degradation behaviors of MX/GG hydrogels were determined by incubating the specimens *in vitro* for 14 days. Briefly, the initial weight (W_i) of each MX/GG hydrogel specimen was first recorded. The specimens were then immersed in PBS solution located on a shaker with its temperature set at 37 °C and a rotating speed of 40 RPM. At specific time points, the hydrogel samples were taken out and their remaining weight was recorded as W_r. The percentage of the degradation (W_d) can be calculated by $W_d = (W_r / W_i) \times 100\%$.

Compression testing

The mechanical fatigue of MX/GG hydrogels was tested by cyclic compression using a rheometer. Briefly, the cyclic compression test, which applied a maximum strain of 50% at a compression speed of 0.067 mm/s, was repeated 10 times for each specimen. The compressive stress-strain measurements of H-XLR and L-XLR from 1.0MX/GG hydrogels were performed using the compression mode of the rheometer. The test was performed with a maximum strain of 80% at a compression speed of 0.067 mm/s.

Cytotoxicity of MXene

The cytotoxicity of the procured MXene was evaluated by introducing it to HUVEC cultures. Culture media with different MXene concentration (0, 0.1, 0.5, 1.0, 2.5, and 5 mg/ml) were prepared. 2 mL cell suspension with a cell density of 2×10^4 cells/mL was added to each well of a 12-well plate, followed by incubation for 12 hours. After that, the culture medium in each well was replaced with medium containing different MXene contents, and the cells were cultured for another 1 to 3 days. The viability of HUVECs was evaluated using the Live/Dead assay, and the fluorescence images were captured using a confocal microscope (Leica MICA, Germany). To evaluate cell proliferation, 200 μl cell suspension with a cell density of 5×10^3 cells/mL was added to each well of a 96-well plate, followed by incubation for 12 hours. 200 μl MXene-containing culture medium was then added to replace the old culture medium, followed by continued culture for 1 and 3 days. Proliferation of HUVECs was tested using CCK-8 assay. To investigate the effects of MXene on cell-matrix interactions, HUVECs were seeded at 2×10^4 cells/ml onto MX/GG hydrogel thin disks with a diameter of ~10 mm. After *in vitro* culture for 3 days, the cell-hydrogel samples were fixed and stained with Hoechst (for nuclei), phalloidin (for F-actin), and antibody against focal adhesion kinase (FAK). For FAK staining, briefly, after adding the monoclonal antibody (Proteintech Group, Inc., China) at a concentration of 1 μg/ml, the sample was incubated at 4 ºC overnight. Afterwards, the cells were labelled with goat anti-rabbit secondary antibody (ThermoFisher) at a concentration of 5 μg/ml. Finally, the cellular cytoskeleton and nuclei were stained with F-actin (Rhodamine Phalloidin, ThermoFisher) and Hoechst (ThermoFisher), respectively. The fluorescence images were captured on the Leica MICA confocal microscope. The mean fluorescence intensity of FAK was calculated using ImageJ.

Finite element analysis (FEA)

FEA was performed to simulate mechanical deformation coupled solvent diffusion for the hydrogel using the commercial software ABAQUS. To model the programmable 3D deformation of the MX/GG hydrogel, a continuum mechanical theory under

isothermal conditions was adopted [7, 8]. For this continuum model, deformation gradient $\boldsymbol{F}$ was multiplicatively decomposed into elastic ($\boldsymbol{F}^e$) and swelling ($\boldsymbol{F}^s$) parts:

$$\boldsymbol{F} = \boldsymbol{F}^e \boldsymbol{F}^s \quad (2)$$

$$\boldsymbol{F}^s = \lambda^s \mathbf{1} \quad (3)$$

$$\lambda^s = (1 + MC)^{1/3} \quad (4)$$

where λ^s is the swelling stretch, M the molar volume of the solvent, and C the fluid concentration.

The free energy (E) that combines the effects of mixing, swelling, and elastic stretching is expressed as:

$$E = \mu^0 C + RTC\left(\ln\left(\frac{MC}{1+C}\right) + \chi\left(\frac{1}{1+MC}\right)\right) + \frac{1}{2}G_s(3(\zeta^2 - 1) - 2lnJ) + J^s\left[\frac{1}{2}G_b(lnJ^e)^2\right] \quad (5)$$

where μ^0 is a reference chemical potential of the fluid, R the gas constant, T the constant temperature under consideration, G_s the shear modulus, G_b the bulk modulus, χ the Flory-Huggins interaction parameter, ζ an effective stretch expressed as:

$$\zeta = \sqrt{\frac{1}{3}(1 + MC)^{2/3} tr\boldsymbol{C}^e} \quad (6)$$

The Cauchy stress (σ) corresponding to the free energy is expressed as:

$$\sigma = J^{-1}\left(2\boldsymbol{F}^e \frac{\partial E}{\partial \boldsymbol{C}^e} \boldsymbol{F}^{eT}\right) = J^{-1}(G\phi^{-2/3}B^e - G\mathbf{1} + J^s G_b(lnJ^e)\mathbf{1}) \quad (7)$$

where ϕ is the polymer volume fraction defined by:

$$\phi \overset{\text{def}}{=} \frac{1}{1+MC} = (\lambda^s)^{-3} \quad (8)$$

Then chemical potential μ, derived from the free energy, can be given as:

$$\mu = \mu^0 + RT(\ln(1 - \phi) + \phi + \chi\phi^2) - MG_b(lnJ^e) + \frac{1}{2}MG_b(lnJ^e)^2 \quad (9)$$

The spatial fluid flux (j) is assumed to be dependent on the spatial gradient of the chemical potential μ and is given by:

$$j = -m\ grad\mu \quad (10)$$

where m is a mobility coefficient.

This continuum theory was numerically implemented via a user-defined element (UEL) subroutine within FEA software ABAQUS/Standard. Two distinct element

formulations were developed:

(1) U1: A 2D plane-strain, 4-node linear isoparametric quadrilateral element.

(2) U2: A 3D 8-node linear isoparametric brick element.

To model differential swelling-driven shape morphing, the MX/GG hydrogel was modeled as a bilayer system, comprising one layer of high crosslinking region (H-XLR) and one hydrogel layer of low crosslinking region (L-XLR). These layers were designed to possess distinct swelling kinetics to simulate the swelling anisotropy for programmable deformation. The interface between the layers was assumed to be bonded perfectly to preclude delamination. For hydrogel strips, it was modelled as 2D plane using the U1 elements under plane-strain assumptions. Conversely, the other 3D architectures (e.g., grippers, floral patterns) were discretized using U2 elements to capture complex volumetric 3D deformations. The model parameters for the FEA simulation were set based on the experimental data of the 1.0MX/GG hydrogels, including the mechanical properties and swelling ratios. The Young's moduli, determined from the nanoindentation and compressive tests, were set as 6 kPa for the H-XLR and 3.5 kPa for L-XLR. The swelling ratios of H-XLR and L-XLR were measured and set as 11.3 and 17.6, respectively. All 4D-printed constructs were first designed in SolidWorks and subsequently exported to ABAQUS for coupled diffusion-deformation analysis, enabling validation of their programmable shape-morphing behaviors under solvent ingress.

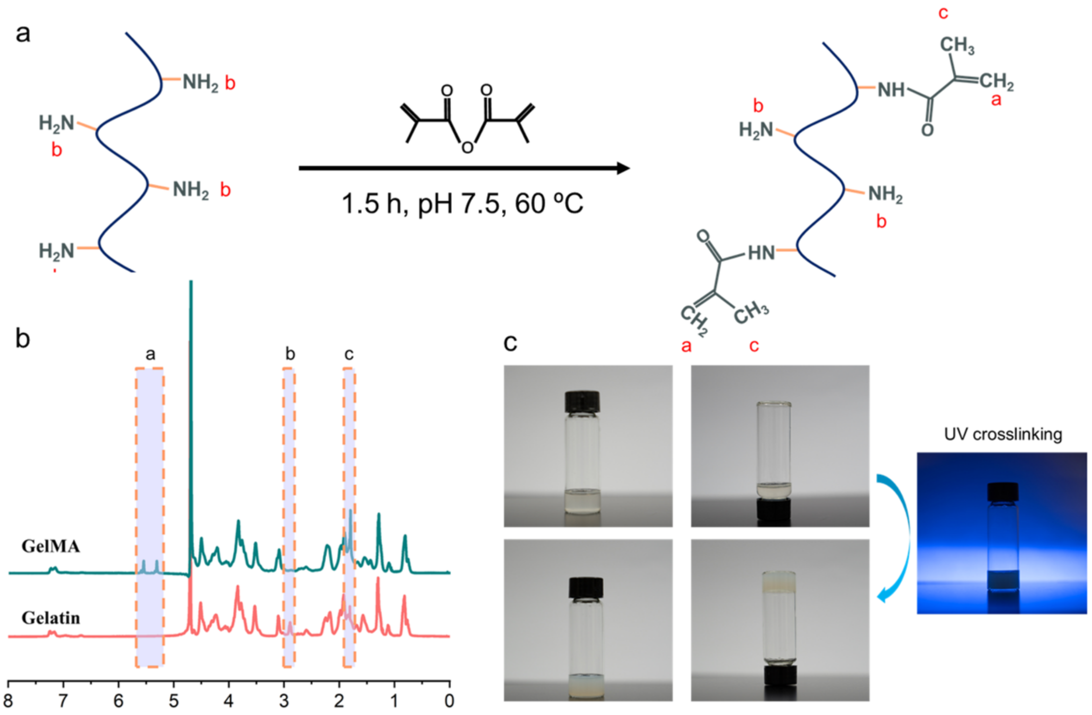

Figure S1. Synthesis and characterization of GelMA. (a) Chemical reaction of GelMA synthesis, (b) ^{1}H NMR spectrum of GelMA showing the representative peaks indicated by a, b and c, (c) pictures showing the successful UV crosslinking of 5 wt.% GelMA.

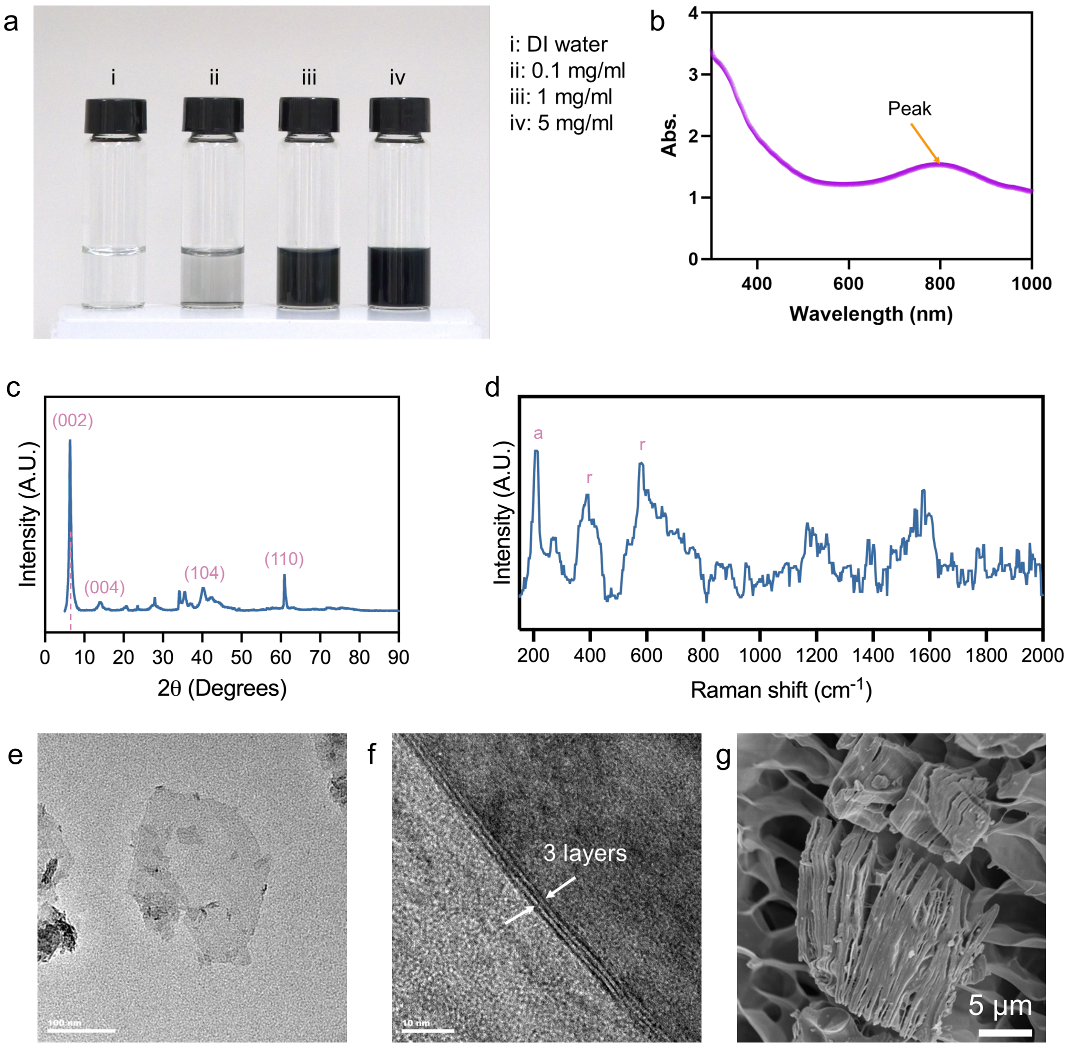

Figure S2. Characterization of MXene. (a) Pictures showing MXene solutions at different concentrations, (b) UV-vis-NIR absorption spectrum, (c) XRD pattern, (d) Raman spectrum, (e, f) TEM images of a MXene nanosheet at (e) low magnification and (f) high magnification, (g) SEM image of MXene nanosheets.

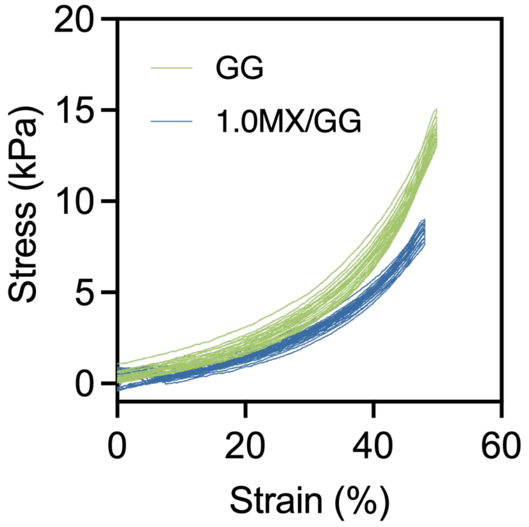

Figure S3. Stress-strain curves for GG and 1.0MX/GG hydrogels during cyclic compression over 10 cycles.

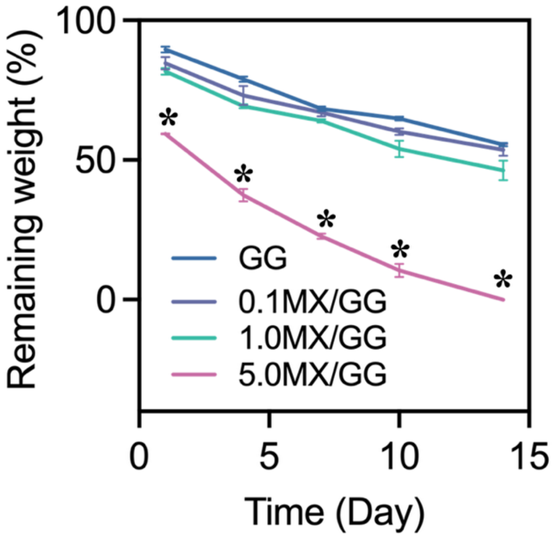

Figure S4. *In vitro* degradation behaviors of GG and MX/GG hydrogels.

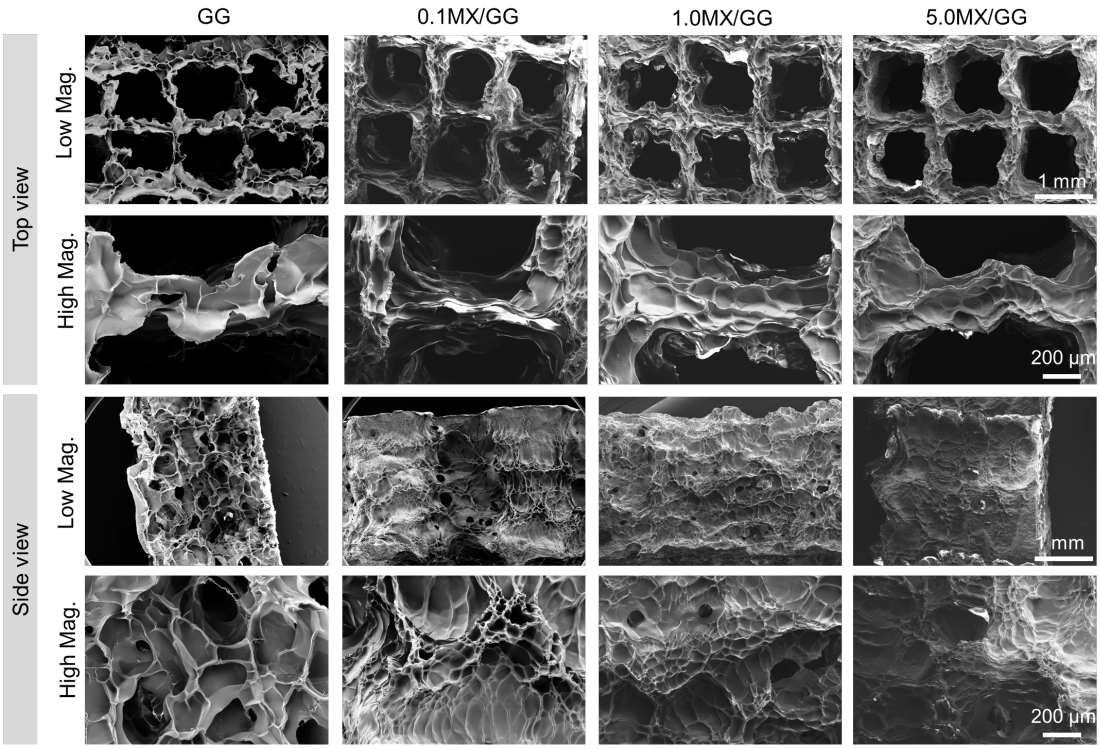

Figure S5. SEM images of 3D printed GG and MX/GG hydrogels. Top views and side views at both low and high magnifications are presented.

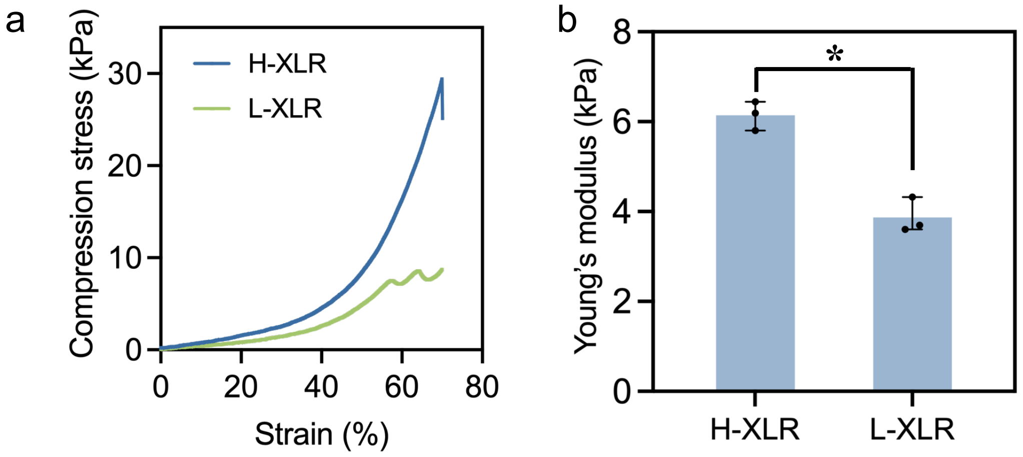

Figure S6. Mechanical properties of H-XLR and L-XLR. (a) Compressive stress-strain curves and (b) corresponding Young's modulus.

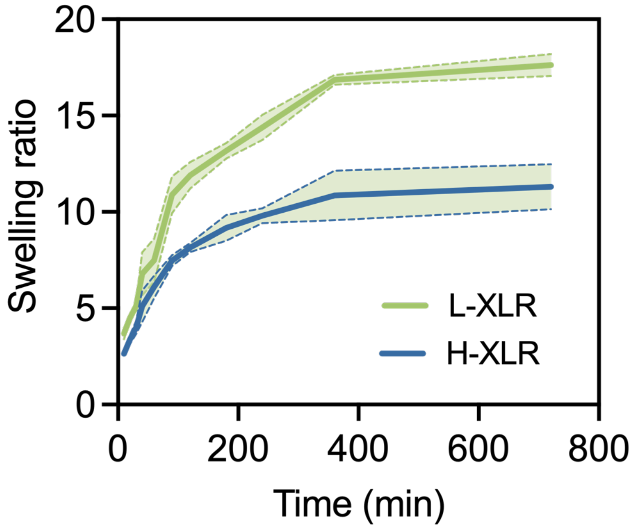

Figure S7. Swelling ratios of H-XLR and L-XLR over time.

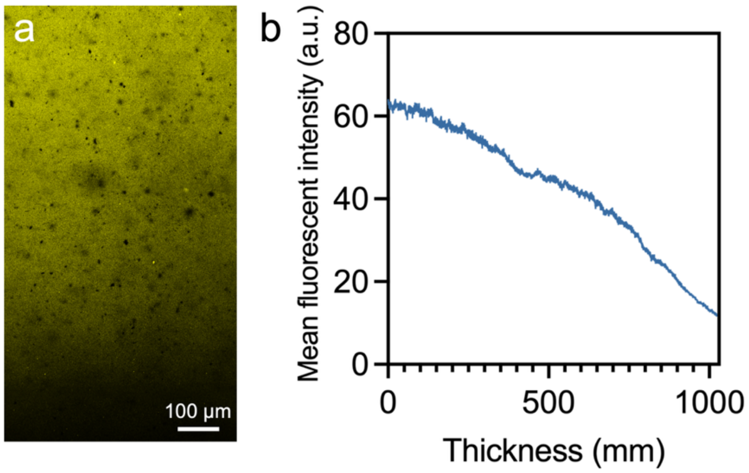

Figure S8. Fluorescence image showing crosslinking gradient across the hydrogel thickness. (a) The fluorescence image and (b) corresponding quantitative analysis of fluorescence intensity across the hydrogel thickness.

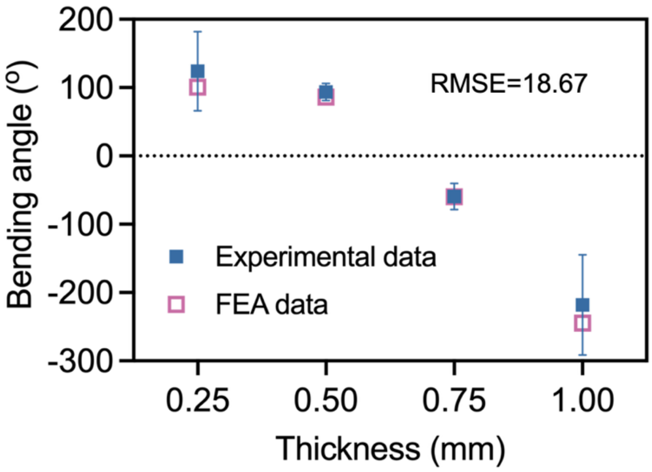

Figure S9. Comparison of the experimental bending angles and FEA-simulated results. The root mean square error (RMSE) is also provided.

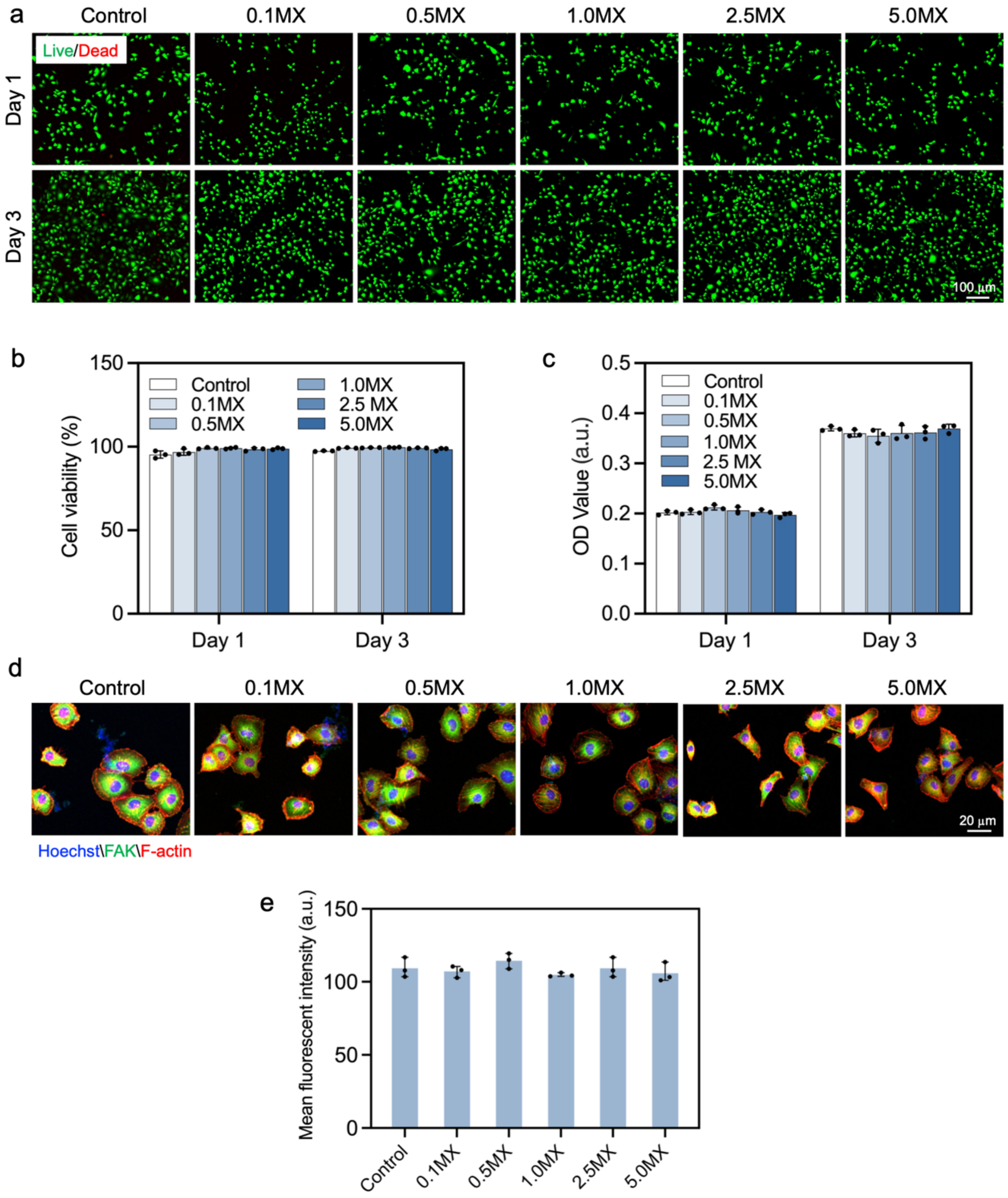

Figure S10. Evaluation of potential cytotoxicity of MXene. (a) Live/Dead staining images for HUVECs after 1 to 3 days of *in vitro* culture with MXene-containing culture medium, with live cells showing green color and dead cells showing red color. (b) Quantitative analysis of live HUVCEs. (c) CCK-8 results showing the proliferation and viability of HUVECs. (d) Immunofluorescence images of the HUVECs seeded onto the MX/GG hydrogels after 3-day *in vitro* culture, and (e) corresponding mean fluorescent intensity of FAK.

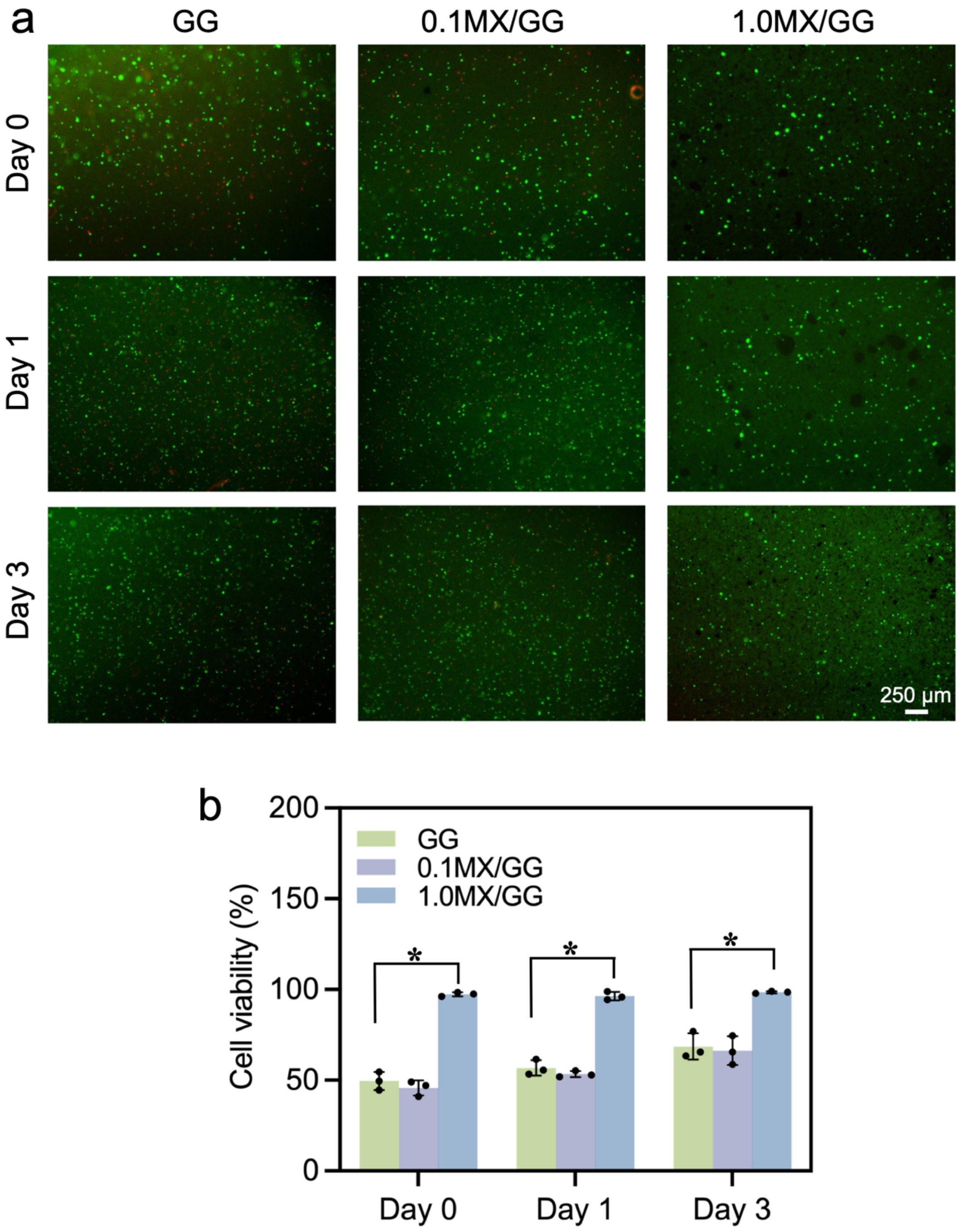

Figure S11. Live/Dead staining for PC12 cell-laden hydrogels. (a) Live/Dead staining images of MX/GG hydrogels containing PC12 cells during 3 days of incubation *in vitro*, with live cells showing green color and dead cells showing red color. (b) quantitative analysis of cell viability.

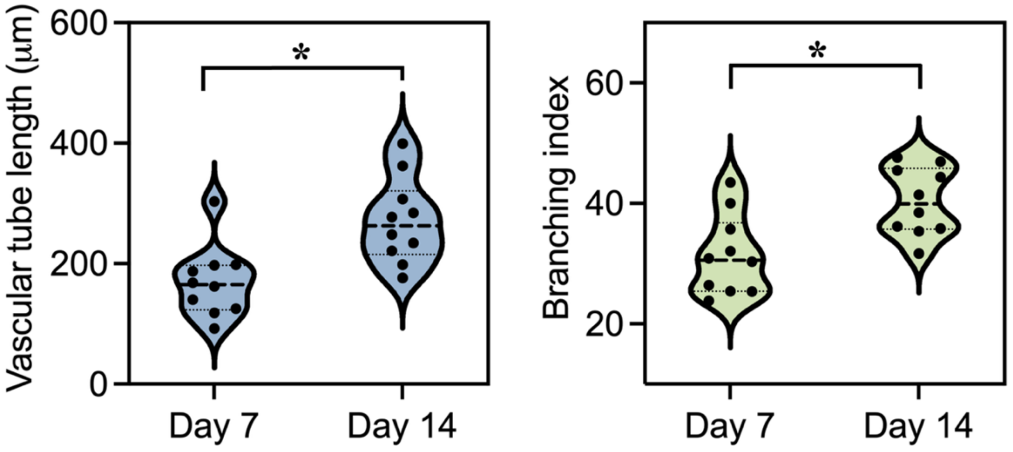

Figure S12. Quantitative analysis of HUVEC tube formation during 14-day *in vitro* culture. (a) Vascular tube length and (b) branching index.

Table S1. Comparison between exiting 4D bioprinting strategies and this work.

4D bioprinting strategy	Bioink composition	4D bioprinter	Method to predict pattern design	External stimuli	Fabrication time	Fabrication complexity	Shape morphing capability	Mechanical properties	Cell viability	Reference
**Crosslinking gradient across the hydrogel thickness direction**	Methacrylate hyaluronic acid (HAMA) or methacrylate alginate (AlgMA) + mesenchymal stem cells (MSCs)	Extrusion-based bioprinting (EBB)	Empirical methods	Water	~12 minutes	Easy, requiring a step to partially dry the scaffold	Unidirectional curvature, limited to single self-folding tubes with a diameter of ~20 μm	__	> 95%	[9]
**The alignment of cellulose nanofiber formed during extrusion leading to uneven swelling within the hydrogels**	Cellulose nanofiber, nanoclay, and N, N-dimethylacrylamide	EBB	Mathematical models based on classical Timoshenko theory	Water	__	Complex smart printing path design, without loading living cells	Mult-directional curvature	Longitudinal pattern: ~1267 kPa Transverse pattern: ~1011 kPa	__	[10]
**Swelling difference between the first layer and second layer**	Bioink 1: Methacrylate silk fibroin + turbinate-derived MSCs Bioink 2: Methacrylate silk fibroin +	Digital light processing	FEA	Water	~13 minutes	Medium, requiring controlling different photo crosslinking degree of the first layer and second layer	Unidirectional curvature	__	__	[11]

	chondrocytes									
**Crosslinking gradient across the thickness of the hydrogels**	GelMA, PEGDA, or oxidized AlgMA microgel + MSCs + UV absorber	EBB	Empirical methods	Water, pH	__	Easy, in some cases requiring photomasks	Unidirectional curvature, requiring photomasks to achieve bi-directional curvature	Young's modulus of oxidized AlgMA microgel: ~0.6 kPa	> 85%	[12-14]
**Swelling difference between the first and second layer**	Bioink 1: tyramine-functionalized hyaluronan (HAT) Bioink 2: alginate/HAT (AHAT) + MSCs	EBB	Empirical methods	Water	__	Easy	Unidirectional curvature	HAT: ~190 Pa (storage modulus), 595 Pa (Yield stress) AHAT: ~203 Pa (storage modulus), 574 Pa (Yield stress)	~75%	[15]
**Opposite charge interaction by immersing anionic GelMA in cationic PLL solution**	GelMA, poly-l-lysine (PLL)	EBB	Empirical methods	Opposite charge interaction	__	Easy	Dimension reduction to ultrathin membrane with a thickness of ~100 µm	__	< 40%	[16]

**Shape memory effect of PLLA-*co*-TMC**	Ink 1: PLLA-*co*-TMC<br>Bioink 2: Gelatin + GelMA + MSCs	EBB	Empirical methods	Temperature	__	Complex, requiring multi-material printing for multi-layer structures	Unidirectional curvature, limited to some simple self-folding structures	Young's modulus: > 90 mPa	> 90%	[17]
**Magnetic actuation**	Ink1: gelatin<br>Ink 2: iron oxide particles + gelatin<br>Ink 3: calcium carbonate + gelatin	EBB	Empirical methods	Magnetic field	__	Medium, involving printing of multiple inks and subsequently solidification of the formed vascular constructs	Unidirectional curvature	Young's modulus: about 0.15 MPa	__	[18]
**Cellular intrinsic force that induces the shape change of the scaffolds during in vitro culture**	Collagen + hyaluronic acid + fibroblasts	Embedded bioprinting	FEA	Cell traction force	__	Medium, the cellular force is hard to control for accurate shape morphing process	Unidirectional curvature	Young's modulus: Day 0: ~0.4 kPa Day 14: ~3.2 kPa	__	[19]
**Thickness-controlled network gradient of different shape morphing domains**	Gelatin + GelMA + MXene + PC12 or HUVECs	EBB	FEA	Water	Several minutes	Easy to operate by simply controlling the thickness of different shape morphing domains	Unidirectional and bidirectional curvatures	H-XLR: 6 kPa; L-XLR: 3.5 kPa	> 90%	This work

Reference:

[1] H. Li, Y.J. Tan, R. Kiran, S.B. Tor, K. Zhou, Submerged and non-submerged 3D bioprinting approaches for the fabrication of complex structures with the hydrogel pair GelMA and alginate/methylcellulose, Additive Manufacturing 37 (2021) 101640.

[2] R. Li, L. Zhang, L. Shi, P. Wang, MXene Ti3C2: An Effective 2D Light-to-Heat Conversion Material, ACS Nano 11(4) (2017) 3752-3759.

[3] S. Boularaoui, A. Shanti, M. Lanotte, S. Luo, S. Bawazir, S. Lee, N. Christoforou, K.A. Khan, C. Stefanini, Nanocomposite Conductive Bioinks Based on Low-Concentration GelMA and MXene Nanosheets/Gold Nanoparticles Providing Enhanced Printability of Functional Skeletal Muscle Tissues, ACS Biomaterials Science & Engineering 7(12) (2021) 5810-5822.

[4] X. Mi, Z. Su, Y. Fu, S. Li, A. Mo, 3D printing of Ti3C2-MXene-incorporated composite scaffolds for accelerated bone regeneration, Biomedical Materials 17(3) (2022) 035002.

[5] H. Rastin, B. Zhang, A. Mazinani, K. Hassan, J. Bi, T.T. Tung, D. Losic, 3D bioprinting of cell-laden electroconductive MXene nanocomposite bioinks, Nanoscale 12(30) (2020) 16069-16080.

[6] M. Mustakeem, J.K. El-Demellawi, M. Obaid, F. Ming, H.N. Alshareef, N. Ghaffour, MXene-Coated Membranes for Autonomous Solar-Driven Desalination, ACS Applied Materials & Interfaces 14(4) (2022) 5265-5274.

[7] S.A. Chester, L. Anand, A coupled theory of fluid permeation and large deformations for elastomeric materials, Journal of the Mechanics and Physics of Solids 58(11) (2010) 1879-1906.

[8] S.A. Chester, L. Anand, A thermo-mechanically coupled theory for fluid permeation in elastomeric materials: Application to thermally responsive gels, Journal of the Mechanics and Physics of Solids 59(10) (2011) 1978-2006.

[9] A. Kirillova, R. Maxson, G. Stoychev, C.T. Gomillion, L. Ionov, 4D biofabrication using shape-morphing hydrogels, Advanced Materials 29(46) (2017) 1703443.

[10] A.S. Gladman, E.A. Matsumoto, R.G. Nuzzo, L. Mahadevan, J.A. Lewis, Biomimetic 4D printing, Nature materials 15(4) (2016) 413.

[11] S.H. Kim, Y.B. Seo, Y.K. Yeon, Y.J. Lee, H.S. Park, M.T. Sultan, J.M. Lee, J.S. Lee, O.J. Lee, H. Hong, H. Lee, O. Ajiteru, Y.J. Suh, S.H. Song, K.H. Lee, C.H. Park, 4D-bioprinted silk hydrogels for tissue engineering, Biomaterials 260 (2020) 120281.

[12] A. Ding, O. Jeon, D. Cleveland, K. Gasvoda, D. Wells, S.J. Lee, E. Alsberg, Jammed Micro-Flake Hydrogel for 4D Living Cell Bioprinting, Advanced Materials n/a(n/a) (2022) 2109394.

[13] A. Ding, S.J. Lee, S. Ayyagari, R. Tang, C.T. Huynh, E. Alsberg, 4D biofabrication via instantly generated graded hydrogel scaffolds, Bioactive Materials 7 (2022) 324-332.

[14] A. Ding, S.J. Lee, R. Tang, K.L. Gasvoda, F. He, E. Alsberg, 4D Cell-Condensate Bioprinting, Small 18(36) (2022) 2202196.

[15] P.J. Díaz-Payno, M. Kalogeropoulou, I. Muntz, E. Kingma, N. Kops, M. D'Este, G.H. Koenderink, L.E. Fratila-Apachitei, G.J.V.M. van Osch, A.A. Zadpoor, Swelling-Dependent Shape-Based Transformation of a Human Mesenchymal Stromal Cells-Laden 4D Bioprinted Construct for Cartilage Tissue Engineering, Advanced Healthcare Materials 12(2) (2023) 2201891.

[16] S.T. McLoughlin, A.R. McKenna, J.P. Fisher, 4D Bioprinting via Molecular Network Contraction for Membranous Tissue Fabrication, Advanced Healthcare Materials 12(27) (2023) 2300642.

[17] S. Chen, J. Li, L. Zheng, J. Huang, M. Wang, Biomimicking trilayer scaffolds with controlled estradiol release for uterine tissue regeneration, Exploration 4(5) (2024) 20230141.

[18] R. Xie, Y. Cao, R. Sun, R. Wang, A. Morgan, J. Kim, S.J.P. Callens, K. Xie, J. Zou, J. Lin, K. Zhou, X. Lu, M.M. Stevens, Magnetically driven formation of 3D freestanding soft bioscaffolds, Science Advances 10(5) (2024) eadl1549.

[19] A. Pramanick, T. Hayes, V. Sergis, E. McEvoy, A. Pandit, A.C. Daly, 4D Bioprinting Shape-Morphing Tissues in Granular Support Hydrogels: Sculpting Structure and Guiding Maturation, Advanced Functional Materials 35(5) (2024) 2414559.
